# Radiation Assessment and Geochemical Characteristics of ^238^U, ^226^Ra, ^232^Th, and ^40^K of Selected Specialized Granitic Occurrences, Saudi Arabia, Arabian Shield

**DOI:** 10.3390/toxics13080612

**Published:** 2025-07-22

**Authors:** Mohamed Tharwat S. Heikal, Aya S. Shereif, Árpád Csámer, Fatma Deshesh

**Affiliations:** 1 Department of Geology, Tanta University, Tanta 31527, Egypt; mohamed.hekal1@science.tanta.edu.eg (M.T.S.H.); aya.salah@science.unideb.hu (A.S.S.); 2 Department of Mineralogy and Geology, University of Debrecen, 4032 Debrecen, Hungary; 3 Cosmochemistry and Cosmic Methods Research Group, University of Debrecen, 4032 Debrecen, Hungary; 4 Department of Geology, Mansoura University, Mansoura 35516, Egypt; fatmadeshesh@mans.edu.eg

**Keywords:** radiation assessment, radioelements, alteration processes, three granitic stocks, Arabian Shield

## Abstract

Between approximately 725 and 518 Ma, a suite of specialized felsic plutons and granitic stocks were emplaced across the Arabian Shield, many of which are now recognized as highly mineralized prospects enriched in rare earth elements (REEs), rare metals, and radioactive elements bearing mineralizations. The current investigation focused on the radiological and geochemical characterization of naturally occurring radionuclides, specifically ^238^U, ^226^Ra, ^232^Th, and ^40^K, within three strategically selected granitic prospects, namely, J. Tawlah albite granite (TW), J. Hamra (HM), and J. Abu Al Dod alkali feldspar syenite and granites (AD). Concerning the radioactivity levels of the investigated granitic stocks, specifically the activity concentrations of ^238^U, ^226^Ra, ^232^Th, and ^40^K, the measured average values demonstrate significant variability across the TW, HM, and AD stocks. The average ^238^U concentrations are 195 (SD = 38.7), 88.66 (SD = 25.6), and 214.3 (SD = 140.8) Bq/kg for TW, HM, and AD granitic stocks, respectively. Corresponding ^226^Ra levels are recorded at 172.4 (SD = 34.6), 75.62 (SD = 25.9), and 198.4 (SD = 139.5) Bq/kg. For ^232^Th, the concentrations are markedly elevated in TW at 5453.8 (SD = 2182.9) Bq/kg, compared to 77.16 (SD = 27.02) and 160.2 (SD = 103.8) Bq/kg in HM and AD granitic stocks, respectively. Meanwhile, ^40^K levels are reported at 1670 (SD = 535.9), 2846.2 (SD = 249.9), and 3225 (SD = 222.3) Bq/kg for TW, HM, and AD granitic plutons, respectively. Notably, these values exceed the global average background levels, indicating an anomalous enrichment of the studied granitic occurrences. The mean radiological hazard indices for each granitic unit generally exceed global benchmarks, except for AEDE_out_ in the HM and AD stocks, which remain below international limits. The geochemical disparities observed are indicative of post-magmatic alteration processes, as substantiated by the interpretation of remote sensing datasets. In light of the significant radiological burden presented by these granitic stocks, it is essential to implement a rigorous precautionary framework for any future mining. These materials must be categorically excluded from uses that entail direct human exposure, especially in residential construction or infrastructure projects.

## 1. Introduction

Almost all construction raw materials and geological derivatives from rock and soil inherently contain variable concentrations of naturally occurring radionuclides, predominantly from ^238^U and ^232^Th decay series, in addition to the radioactive potassium isotope ^40^K. These naturally occurring radionuclides constitute primary sources of both external and internal radiological exposure within inhabited structures. External exposure arises from the emission of gamma radiation directly from the building materials. In contrast, internal exposure predominantly results from the inhalation of radioactive noble gases like ^222^Rn, a decay progeny of ^226^Ra and ^220^Rn, derived from ^224^Ra. The subsequent decay of these isotopes into short-lived daughter nuclides emits high-energy alpha particles, which pose a significant hazard to the respiratory epithelium upon inhalation.

The specific activities of ^238^U, ^226^Ra, ^232^Th, and ^40^K in construction raw materials and related products are predominantly governed by their geological provenance, geographical context, and the inherent geochemical composition of the source materials [1]. Within the ^238^U decay series, the segment of the decay chain commencing from ^226^Ra is regarded as the most radiologically significant. Consequently, ^226^Ra is frequently employed as a radiological proxy for ^238^U [2]. It is imperative to ascertain the dose limits for public exposure and to quantitatively assess the levels of ambient natural radiation emanating from the ground, air, water, foods, and building interiors. Such evaluations are essential for accurately estimating human exposure to naturally occurring radioactive sources [3]. The radiological implications of naturally occurring radioactivity primarily stem from external gamma-ray exposure and the internal irradiation of lung tissues, which arises from the inhalation of radon gas and its short-lived radioactive progeny [1].

The concentration of natural radioelements within building materials and their constituents plays a pivotal role in evaluating radiological exposure levels among the general population, particularly given that individuals typically spend about 80% of their time indoors [1]. The globally averaged indoor absorbed gamma dose rate, attributed to terrestrial sources of radioactivity, has been estimated to be 70 nGy/h [4], whereas the world mean annual effective dose equivalent attributable to gamma radiation exposure from construction materials is estimated to be approximately 0.4 mSv [5]. To mitigate the potential health hazards associated with elevated concentrations of naturally occurring radionuclides in construction materials, regulatory measures and usage restrictions must be implemented, particularly in residential buildings, according to the recommendation of IAEA in its Basic Safety No. 112 [2].

Certain felsic igneous rocks, particularly specialized varieties of granite, play a pivotal role as repositories for naturally occurring radionuclides, owing to their crystallization during the terminal phases of magmatic differentiation. The geochemical affinity of these rocks is further amplified by the presence of accessory minerals, such as zircon, apatite, thorite, uraninite, and monazite, which serve as effective hosts for radioisotopes of U, Th, and K, incorporating them into their crystalline lattices through isomorphous substitution and lattice entrapment mechanisms [6,7,8,9].

**Figure 1:**
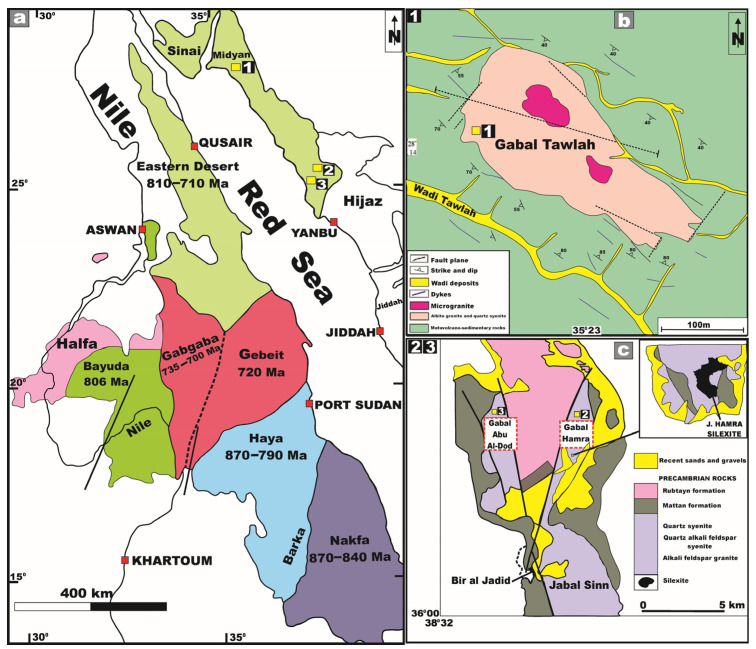
(**a**) Tectonostratigraphic terrane map of the Arabian–Nubian Shield reproduced from [14]. 2003, Geol. Soc. London, Spec. Publ. Note the location of the three studied prospects (yellow squares); (**b**) 1 J. Tawlah prospect (TW) reproduced from [11], 2022, J. African Earth Sci.; and (**c**) 2 J. Hamra prospect (HM) and 3 J. Abu al Dod prospect (AD), Saudi Arabia, Arabian Shield, reproduced from [15], 2007, Cent. Eur. Geol.

It is well known that the Arabian–Nubian Shield (ANS) has several mineralized terranes and regions (e.g., [10,11,12,13]). Midyan Terrane constitutes one of the most economically significant metallogenic provinces within the northwestern sector of the Arabian Shield (Figure 1). It is notably enriched in polymetallic mineralization, comprising Mo, Nb, REEs, Sn, Ta, U, W, and Zr. This terrane also hosts numerous felsic intrusive bodies, predominantly consisting of younger metaluminous to peralkaline granitic suites, ranging from alkali to alkali feldspar granite varieties (Figure 1).

The investigated granitic stocks—Tawlah (TW), Hamra (HM), and Abu Al Dod (AD)—are recognized as notable representatives of the Ghurayyah-type mineralization, genetically linked to the Midyan Terrane. Genetically, they are related to post-collision, within-plate, rare-metal-bearing granites [11]. These stock bodies exemplify extremely enriched intrusions in heavy REEs and Y (e.g., [10,16]). Accordingly, given the significant economic potential of these prospects, it is imperative to undertake a comprehensive assessment and quantitative evaluation of their intrinsic radioactivity levels through the deployment of advanced radiometric analytical techniques.

To date, the present study constitutes the inaugural in-depth endeavor to rigorously evaluate the radiological footprint of naturally occurring radionuclides within these granitic prospects, an undertaking of critical importance for understanding potential environmental repercussions impacting both the general populace and occupational exposure. Moreover, geochemical investigations are concurrently undertaken to elucidate the provenance and such controls governing the enrichment and mobilization of these radioelements.

## 2. Geology of the Study Area

J. Tawlah granitic stock (TW) represents a modestly sized, post-collisional, intraplate granitoid stock enriched in rare-metal and thorium constituents. It encompasses an area of approximately 11 km^2^ and is geographically situated in northwest Saudi Arabia at latitude 28°14′28″ N and longitude 35°23′30″ E (Figure 1). It lies some 110 km west of Tabuk in the north–central part of the Midyan terrane (Figure 1). Geochronological investigations based on isotopic systematics of Sr and Nd (^87^Sr/^86^Sr and ^143^Nd/^144^Nd) yielded an emplacement age of 577 ± 4 Ma [17]. Recently, novel geological insights into J. Tawlah stock has been documented for the first time by Gahlan et al. (2022) [11]. J. Tawlah represents a significant focus of rare-metal enrichment, exhibiting considerable potential as a prospective source of Nb, Zr, Y, Th, Ta, and Sn (e.g., [18,19]). Petrographically, the Tawlah intrusive body is predominantly composed of albite granite, with subordinate occurrences of quartz syenite.

The albite granite exhibits pronounced mineralization, characterized by an elevated modal albite content and notably higher Nb/Ta ratios [11]. From a structural perspective, the spatial correlation between fault networks and shear zones, alongside the distribution of mineralizations, offers compelling evidence for the tectonically controlled emplacement.

**Figure 2:**
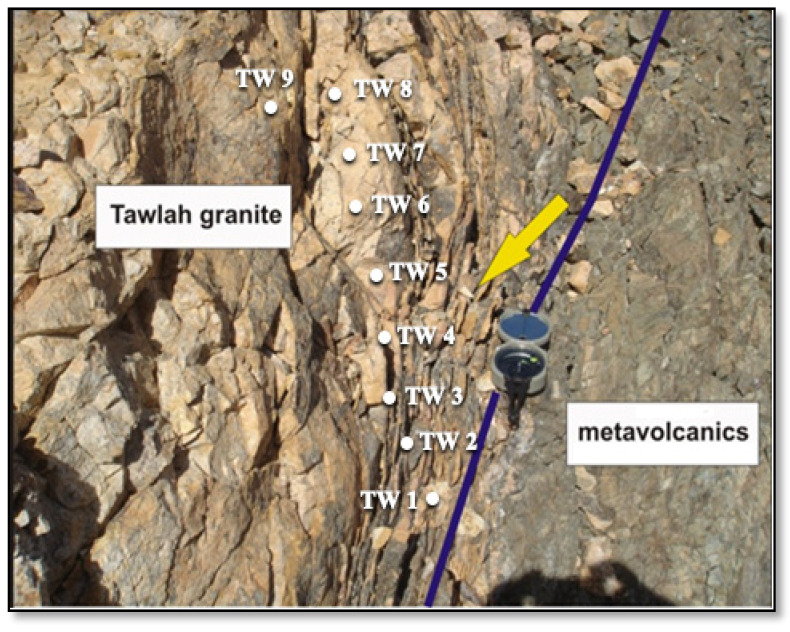
An exposure of J. Tawlah granitic stock (TW) showing intrusive contact against the oldest country rocks of metavolcanics and volcanoclastic. Note highly fractured and shearing (arrow) trending N–S and NW–SE, where samples were taken along shear zones. Field photograph captured by the authors in 2012.

It is noteworthy that the Najd Fault Zone offers critical insights into the genetic linkage between regional fault systems and the emplacement of REEs-Nb-Ta-Th mineralizations. The spatial distribution of these mineralized zones exhibits strong structural control, aligned along the principal tectonic trends, WNW–ESE, NW–SE, and ENE orientations (Figure 2). Furthermore, the reactivation of the NW-oriented Najd fault system, influenced by tertiary extensional rift tectonics, appears to have played a pivotal role in localizing and enhancing mineralization processes within the J. Tawlah alkaline granite suite [11].

The J. Tawlah stock exhibits a distinctly rectangular morphology, characterized by short, steep-sided ridgelines situated along the NNE margin of Wadi Tawlah. Field observations reveal that J. Tawlah granites are emplaced in sharp intrusive contact with the surrounding country rocks, which predominantly comprise ancient metavolcanic sequences interbedded with volcaniclastics (Figure 2). Furthermore, the region is structurally dissected by an intricate network of brittle deformation features, including prominent fracture systems and shear zones oriented predominantly along N–S and NW–SE trends (Figure 2).

Multiple N–S-oriented on-foot geological traverses were systematically undertaken across the granitic stock in varying directions to delineate the nature of the exposed lithologies, identify structural fabrics, perform in situ radiometric measurements, and collect representative rock samples from each point along these profiles (Figure 2).

**Figure 3:**
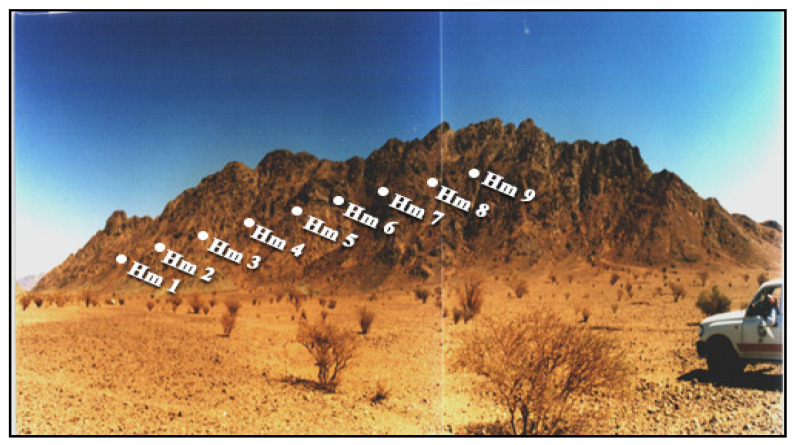
General view of Jabal Hamra granite (HM). Samples were collected across the shear zones of the same stock. Field photograph captured by the authors in 2012.

J. Hamra is geographically situated at the coordinates 26°04′ N and 38°36′ E, in the northeastern sector of the Arabian Shield (Figure 1). This plutonic body is predominantly composed of alkali feldspar granites and silexite (siliceous rock), with subordinate occurrences of quartz syenites (Figure 3). J. Hamra, dated at approximately 538 Ma [17], encompasses an area of about 6.5 km^2^.

J. Hamra is hypothesized to represent a fault-bounded intrusive body emplaced within a substantial succession of metamorphosed volcano-sedimentary and pyroclastic rocks [20]. Field data reveal a complex network of brittle deformation features, including prominent fracture systems and shear zones exhibiting predominant NE and NW orientations.

From a mineralogical and economic perspective, J. Hamra exhibits substantial enrichment in rare metals, including Ta, Nb, Sn, and REEs. Furthermore, the area is characterized by anomalously elevated levels of natural radioactivity [16,20].

Regarding J. Abu Al Dod stock, it encompasses an area of approximately 10 km^2^ and is situated close to J. Hamra (Figure 1). Intervening between these two plutonic bodies is a substantial sequence of metamorphosed volcanoclastic units, structurally manifested as a fault-bounded horst block [20].

**Figure 4:**
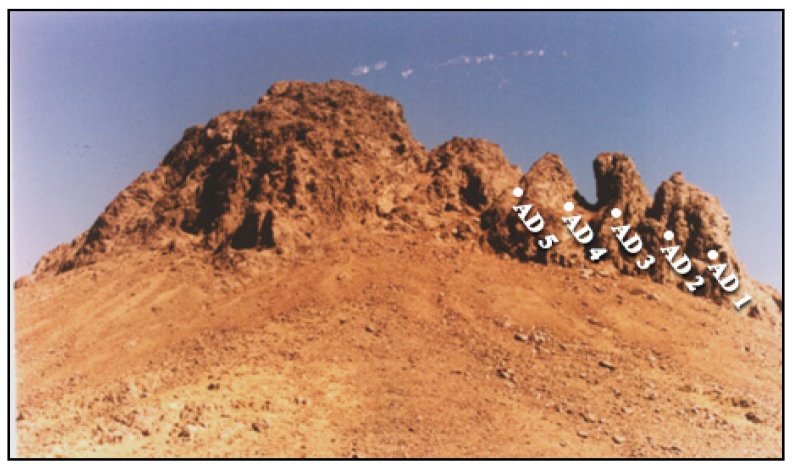
General view of Jabal Abu Al Dod granite (AD). Samples were collected across the shear zones of the stock. Field photograph captured by the authors in 2012.

J. Abu Al Dod exhibits a rugged and dissected topography, intricately transected by numerous shear zones and fractured systems predominantly oriented in a NW direction (Figure 4). It is predominantly composed of alkali feldspar granites, with subordinate occurrences of quartz syenites. The investigated stock exhibits significant enrichment in rare metals and REEs, coupled with notable anomalous radioactivity [20].

## 3. Materials and Methods

### 3.1. Sample Collections

**Figure 5:**
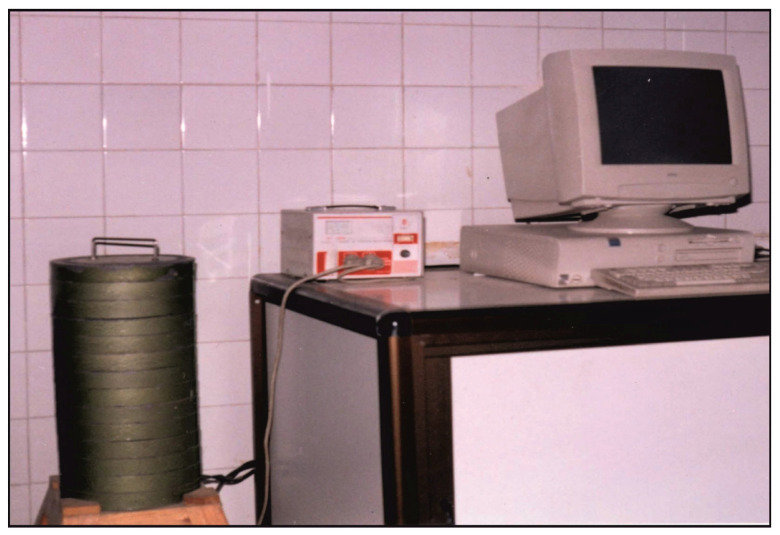
Na-I detector. Source: Research Division. Geochem. Exp. Dept. NaI Laboratory, Nuclear Materials Authority (NMA), Egypt.

A total of 23 representative rock samples were systematically collected from the principal lithological units of J. Tawlah (9 samples), J. Hamra (9 samples), and J. Abu Al Dod (5 samples). These samples were acquired along detailed on-foot geological traverses, specifically in proximity to and/or aligned with prominent shear zones and fracture systems within the three investigated plutonic bodies (Figure 2, Figure 3 and Figure 4). In situ field radioactivity measurements were exclusively conducted across the J. Tawlah granitic stocks. Conversely, lab radiometric analyses for rock samples from all three investigated stocks were systematically performed using a high-resolution NaI (Tl) scintillation spectrometric detector (Figure 5).

### 3.2. Field Radioactivity Measurements

Field radiometric surveys were systematically conducted across various sampling sites within J. Tawlah. Additional measurements were carried out across structurally significant features, including shear zones, quartz veins, and pegmatitic bodies. The instrument employed for this investigation was a portable gamma-ray scintillometer (Model G.R-101A), manufactured by GeoMetrics, San Jose, CA, USA, 1980 [21]. It operates at a voltage of 3 V DC with a current of 100 mA. Ground-based radioactivity was recorded in terms of total gamma-ray counts per second (cps), providing a quantitative assessment of the natural radiation intensity across the surveyed localities. The measurements were acquired at an approximate spatial interval of 3 m between sampling points.

The field radiometric investigation at J. Tawlah provides the following findings:

(1) The medium-grained albite granite, constituting the predominant lithology within J. Tawlah exhibits markedly elevated radioactivity levels, with scintillometric readings ranging from 1800 to 2300 cps, and an average of approximately 1900 cps.
(2) Quartz microsyenite, ranking second in abundance after the medium-grained albite granite, is characterized by comparatively subdued radioactivity levels, typically ranging between 1000 and 1400 cps.
(3) The deformed albite granite exhibits pronounced shearing and pervasive silicification, resulting in markedly elevated gamma field radioactivity, with readings peaking at approximately 3200 cps and an average value of around 2500 cps.
(4) Shear zones, along with several quartz veins, also exhibit notably elevated levels of radioactivity, typically ranging between 1900 and 2500 cps.

### 3.3. Gamma-Spectrometric Measurements

The quantification of uranium and thorium activity concentrations in ore and lithological samples predominantly depends on precise radiometric techniques, notably through the deployment of gamma-ray spectrometry utilizing multi-channel analyzers (Figure 5). In the present investigation, radiometric analyses were performed using NaI (Tl) scintillation detectors sourced from GeoMetrics, located in San Jose, CA, USA. operated within the Radiation Protection Department laboratory of the Nuclear and Radiological Regulatory Authority (Egypt). The NaI (Tl) scintillation detector, while exhibiting relatively limited energy resolution, is distinguished by its superior detection efficiency (Figure 5). This high intrinsic efficiency facilitates the rapid and accurate determination of radionuclide activity concentrations of ^238^U, ^226^Ra, ^232^Th, and ^40^K in diverse geological matrices, including rocks, soils, and sand samples. The results depend on the accuracy of the energy calibration procedure, which takes into account the possibility of interference of the individual nuclides within each peak region [22].

The gamma-ray spectrometry system comprises a Bicron scintillation detector, manufactured by Bicron in Newbury, OH, USA, equipped with a 76 × 76 mm NaI (Tl) crystal hermetically sealed and coupled to a photomultiplier tube housed in an aluminum casing. The detector is protected from ambient radiation by a chamber made of lead bricks and from induced X-rays by a cylindrical copper shield (0.6 cm thick). A 5 cm thick lead cover is then placed over the detector. The detector relates to the Tennelec high-voltage power supply with digital HV display and Nuclear Enterprises main shaping amplifier. A laser printer and Nuclease PCA-8000 computerized, 8192 multichannel analyzers with a color graphical spectrum display and advanced technical operation features are also connected to the detector (Figure 5).

The measurements were carried out in cylindrical plastic sample containers with a volume of 212.6 cm^3^, an average diameter of 9.5 cm, and a height of 3 cm. The measurement of radionuclides is based on the selection of three energy regions of interest (ROIs) representing ^234^Th, ^214^Pb, ^212^Pb, and ^40^K for U, Ra, Th, and K, respectively. It is assumed that the samples are in secular equilibrium, which allows for the indirect determination of the parent radionuclides’ activity concentrations through their gamma-emitting progeny [23]. The gamma transition (92.6 keV) from ^234^Th decay was used to determine the activity concentration of ^238^U, while the gamma transitions (352 and 238.6 keV) from the ^214^Pb and ^212^Pb decay were used to determine the activity concentrations of ^226^Ra and ^232^Th, respectively. The activity concentration of ^40^K was measured directly from the 1460.8 keV peak energy. Uranium and thorium are not gamma-emitters and consequently, they are measured indirectly using their γ-ray emitting daughters, ^234^Th and ^212^Pb, respectively.

The following equation was used to obtain the activity which was then converted to the activity concentration in Bq/kg for each sample [24].

A_c_ (Bq/kg) = C_n_/P_Ɣ_MƐK

where A_c_ is the activity concentration for the element in the sample determined by Bq/kg, (C_n_) is the net count rate under the corresponding peak, (P_Ɣ_) is the absolute transition probability of the specific gamma-ray, (M) is the mass of the sample (kg), Ɛ is the detector efficiency at the specific gamma-ray energy, and (K) is the correction factor for the nuclide decay from the time of sampling to counting. Numerous factors affect the quality of the results obtained, including: (i) the size and energy resolution of the scintillation detector, (ii) geometry and mass of the sample, (iii) performance of the multichannel analyzer and its operation stability, (iv) shielding of the detector, (v) selection of specific detected gamma-ray energies, (vi) time of measurements, (vii) quality and reliability of the standards used, and (viii) the data processing technique.

The process of the radionuclides measurement by the NaI (Tl) detector is carried out after two main steps: (a) energy and (b) sensitivity calibrations.

a. Energy calibration

The permanent calibration was performed with the radioactive calibration sources ^137^Cs (661.6 keV) and ^57^Co (122.1 keV) to ensure that the device accurately records the gamma radiation energy of the radioactive elements as follows: Calibration started with the ^137^Cs source (gain adjustment) and then with the ^57^Co source (zero adjustment). The ^137^Cs source was used repeatedly as the minimum procedure.

b. Sensitivity calibration

Since the gamma-ray spectrometer is used for geochemical prospecting, it must be calibrated in terms of isotopic sensitivity (i.e., converting counts per unit of time into an isotopic concentration in parts per million or percent). Three synthetic standard sources (geological reference materials) were used for the calibration. These standards were prepared using a series of certified reference samples with specific U, Th, and K concentrations obtained from the International Atomic Energy Agency (IAEA), Vienna, Austria. The sensitivity of the instruments used was determined by measuring the three standards twice, each for 1000 s, and then averaging the gross counts collected in the selected ROIs. The count rates were normalized per unit mass and adjusted for background count rates in the ROIs. The corrected values were entered into the computer program “ANALYSIS”, which was developed for laboratory gamma-ray spectrometry of geological materials [25]. The final result is a matrix of instrument sensitivity values, each of which is represented by the count rate per unit mass of the geologic material measured and per unit of the radioelement concentration. These sensitivity values of the equipment are used as a reference in the analysis of the unknown rock samples. The lower limit of detection of ^238^U, ^226^Ra, ^232^Th, and ^40^K is 44.12, 32.22, 42.33, and 1028 Bq/kg, respectively. The expected measurement errors usually range between 7 and 12%.

### 3.4. Remote Sensing Data

Landsat-9 satellite data were rigorously processed utilizing ENVI software (version 5.1) in conjunction with ArcGIS 10 to delineate the lithologies enriched in radionuclides and to detect associated hydrothermal alteration zones within the investigated area. The digital image processing workflow yielded a suite of high-resolution outputs, including false-color composite (FCC) imagery and band ratio transformations, all rendered at a detailed cartographic scale of 1:50,000. These remote sensing products significantly enhanced the geological interpretation by revealing key lithological contacts, structural features, and spatial patterns of alteration, thereby facilitating the accurate delineation of radionuclide-bearing formations and contributing to a more comprehensive understanding of the region’s tectono-radiometric framework.

## 4. Petrographic Inspection

A concise petrographic description of the three principal investigated granitic stocks was conducted utilizing a high-resolution Zeiss research-grade polarizing microscope. The dominant rock type of J. Tawlah is albite granite, whereas J. Hamra predominantly comprises alkali feldspar syenite and silexite (siliceous rock). On the other hand, the main rock type at J. Abu Al Dod is alkali feldspar granite. Megascopically, these granitoids exhibit medium-to-coarse-grained rocks. The color index varies noticeably among the stocks, J. Tawlah displays hues ranging from milky white to greyish-white, while those from J. Hamra and J. Abu Al Dod are typified by vivid reddish to pinkish-red tones.

### 4.1. Albite Granite

**Figure 6:**
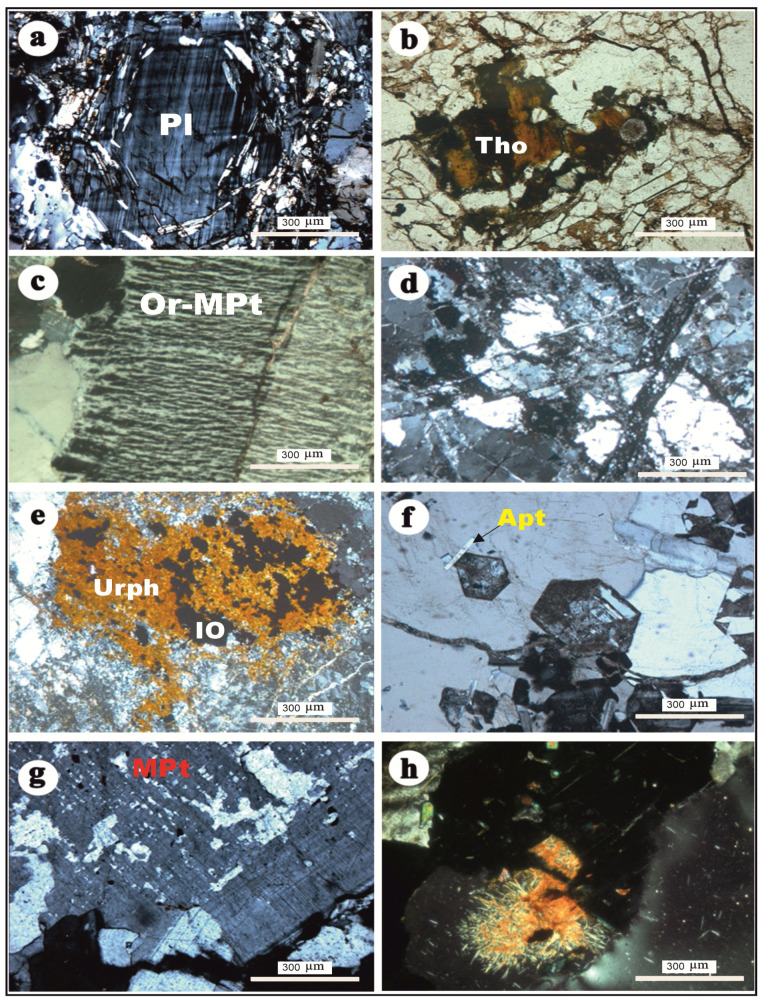
(**a**) Typical snowball texture in J. Tawlah albite granite, XPL; (**b**) thorite (Tho) crystal as radioactive mineral rimmed by iron oxides (IO) in J. Tawlah albite granite, PPL; (**c**) orthoclase–microperthite (Or-MPt) of flame type invaded by quartz crystals in alkali feldspar syenite of G. Hamra pluton, XPL; (**d**) highly resorbed quartz and alkali feldspar in silexite, J. Hamra pluton (note micro reverse fault crosscutting all mineral constituents), XPL; (**e**) dense patch of uranophane (Urph) within alkali feldspar granite, G. Hamra pluton, PPL; (**f**) well-developed crystals of apatite (Apt) in alkali feldspar granite, Hamra stock, XPL; (**g**) microperthite (MPt) of flame and vein types in alkali feldspar granite, J. Abu Al Dod, XPL; and (**h**) typical radiation due to radiogenic effects related to secondary uranium mineral in alkali feldspar granite, J. Abu Al Dod, XPL.

Albite granite represents the main rock type of the J. Tawlah stock. These granitoids are characteristically medium-grained, hypidiomorphic, and exhibit various shades of whitish coloration. Petrographically, the medium-grained albite granite is composed primarily of albite, quartz, and microcline and is notably distinguished by its classic snowball texture (Figure 6a). Plagioclase (Pl) feldspar of albite composition (An_5_–An_10_) is the dominant mineral with an average of 45% of the rock mode (in a few samples, it decreases to 30% of the rock mode). Albite crystals are fine-grained, subhedral, with a prismatic to lath-like form. The albite laths exhibit interpenetrating and interlocking textures (Figure 6a), while in other samples, they display subparallel alignment around the coarse, slightly elongated, and deformed quartz. Accessory and opaque minerals are conspicuously abundant, constituting approximately 5–10% of the rock’s modal composition, and in certain samples, their proportion may reach up to 15%. These minerals include zircon, apatite, xenotime, and a substantial presence of thorite, which alone may constitute up to 5% of the total mineral assemblage (Figure 6b).

### 4.2. Alkali Feldspar Syenite

The alkali feldspar syenite represents the main rock type of the J. Hamra pluton. These rocks exhibit a predominantly equigranular, fine-to-medium-grained texture and are characterized by their distinctive pink to reddish hues. Notably, several specimens display a porphyritic texture, particularly pronounced within the roof facies. These rocks are predominantly composed of alkali feldspar minerals, notably orthoclase and flame-type microperthite (Figure 6c), along with quartz, which together constitute approximately 90–95% of the modal mineralogy. Minor lath-shaped albite crystals are also present. The mafic components have been extensively pseudomorphed by pervasive iron oxides, secondary quartz, and calcite, reflecting intense post-magmatic alteration. Accessory minerals include zircon, fluorite, allanite, and concentrated patches of uranophane, in addition to well-formed apatite crystals (Figure 6e,f).

### 4.3. Quartzolite (Silexite)

Quartzolite or silexite (siliceous rock) here is referred to as an intrusive igneous rock with a high quartz content (higher than 90%) [26]. In the present study, the term of hematosilconization is adopted to describe a dual alteration process involving pervasive silicification coupled with hematitization. This integrated alteration phenomenon results in the formation of a distinctive rock type, herein referred to as hematite–silica-altered rock. The protolith of these altered rocks is inferred to have undergone uranium-bearing fluid interactions, whereby uranium (U) is believed to have been introduced concurrently with the hematosilconizing solutions. Notably, uranium may have been adsorbed onto or finely disseminated in iron oxide phases, which act as efficient geochemical traps during the alteration process.

In the early stage of hematosilconization, the partially hematosilconized quartz alkali feldspar syenite consists of alkali feldspar and quartz and abundant secondary minerals, which include quartz, hematite, and calcite, forming vein-like hematite–silica-altered rock (Figure 6d). The alkali feldspars are highly clouded and stained with a pale reddish-brown color. The crystals are also cut by thin, delicate dendritic veinlets of iron oxides, and a few medium-grained crystals are delineated with a thin, clear albite rim (Figure 6d). Quartz crystals are strongly deformed with the development of undulose extinction. The crystals are highly corroded by the solution that precipitated very-fine-grained micro- to cryptocrystalline secondary quartz (groundmass). In the highly hematosilconized rock, alkali feldspar crystals are completely destroyed with the precipitation of secondary quartz and iron oxides. Hematite–silica-altered rock is a fine-grained rock of a yellowish brown to reddish brown and dark blackish gray. The medium-grained quartz crystals (with a 4 mm average diameter) are highly strained and fractured, corroded, and embayed.

### 4.4. Alkali Feldspar Granite

This rock type is extensively developed within the J. Abu Al Dod plutonic stock. Petrographically, the rock is predominantly composed of K-feldspar and quartz, with subordinate amounts of plagioclase feldspar (Figure 6g). The mineral assemblage exhibits a coarse-grained, equigranular, and hypidiomorphic texture. K-feldspar is represented by both microcline and orthoclase–microperthite, exhibiting well-developed flame-to-vein-type perthitic intergrowths (Figure 6g). These crystals are coarse-grained, subhedral, and typically display a tabular habit. Notably, several grains are stained with a pale reddish-brown hue, attributed to secondary alteration by finely disseminated iron oxides intermingled with clay minerals.

Quartz is present in two different types: The primary variety occurs as medium-to-coarse-grained anhedral crystals, commonly exhibiting undulose extinction and strain-induced deformation features. The secondary variety comprises very fine-grained micro-to-cryptocrystalline aggregates, typically formed through pervasive silicification processes. Additionally, iron oxides are conspicuously abundant, occurring as discrete veinlets and irregular black patches, often intimately associated with the secondary quartz. Accessory minerals are less abundant than those found in G. Hamra syenite. They include zircon, apatite, and secondary uranium minerals (Figure 6h).

## 5. Key Findings and Discussion

### 5.1. Distribution of Radionuclides (^238^U, ^226^Ra, ^232^Th, and ^40^K)

**Figure 7:**
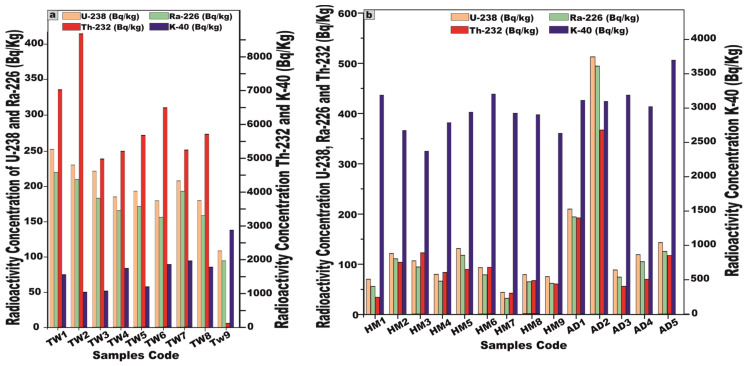
Plot of the activity concentrations of radionuclide elements ^238^U, ^226^Ra, ^232^Th, and ^40^K (Bq/kg) (**a**) for J. Tawlah albite granites (TW), (**b**) for J. Hamra Alkali feldspar granites stock (HM), and Abu Al Dod granitic pluton (AD), Saudi Arabia, Arabian Shield.

The activity concentrations of radionuclides were meticulously quantified in 23 representative samples extracted from the granitic stocks of the Arabian Shield, Saudi Arabia. The dataset encompasses nine samples from J. Tawlah albite granite (TW), nine samples from J. Hamra stock (HM), and five samples from J. Abu Al Dod (AD) (Table 1 and Figure 7a,b). These specimens were meticulously curated for laboratory analyses, facilitating an in-depth radiometric assessment to ascertain their precise activity concentrations of ^238^U, ^226^Ra, ^232^Th, and ^40^K radionuclides.

For J. Tawlah albite granite (TW) samples, the ^238^U activity exhibited a substantial variation, ranging from 108.6 to 252.8 Bq/kg, with a mean value of 195.3 Bq/kg. ^226^Ra activity spanned 94.6 to 220.1 Bq/kg, yielding an average of 172.4 Bq/kg. Notably, the ^232^Th activity concentration demonstrated an extensive fluctuation, ranging from 111.2 to 8677.3 Bq/kg, with an elevated average concentration of 5453.8 Bq/kg, signifying potential thorium enrichment. Furthermore, the ^40^K activity oscillated between 1028 and 2870 Bq/kg, with an average of 1670 Bq/kg, reflecting the potassium-bearing mineral composition of the granite (Table 1 and Figure 7a).

Regarding the analyzed Hamra alkali feldspar granite (HM) samples, the radionuclide ^238^U displayed activity concentrations ranging from 44.12 to 131.2 Bq/kg, with a mean value of 88.7 Bq/kg. Similarly, ^226^Ra concentrations fluctuated between 32.22 and 117.7 Bq/kg, averaging 75.6 Bq/kg. The concentration of ^232^Th spanned from 34.4 to 121.8 Bq/kg, yielding a mean value of 77.16 Bq/kg. Moreover, the concentration of ^40^K varied from 2374 to 3199 Bq/kg, with an average of 2846.2 Bq/kg (Table 1 and Figure 7b).

Concerning the investigated Abu Al Dod granite (AD) samples, the concentration of ^238^U ranged from 88.12 to 512.11 Bq/kg, with a mean value of 214.3 Bq/kg, while ^226^Ra levels fluctuated between 74.12 and 493.8 Bq/kg, averaging 198.4 Bq/kg. Similarly, the activity concentration of ^232^Th spanned from 55.6 to 366.7 Bq/kg, giving an average of 160.2 Bq/kg. Meanwhile, ^40^K exhibited concentrations ranging from 3023 to 3700 Bq/kg, with a mean value of 3225 Bq/kg (Table 1 and Figure 7b).

These subsequent findings unequivocally suggest that all the granitic samples exhibit elevated uranium (^238^U) and radium (^226^Ra) concentrations surpassing the globally recognized baseline [1,5]. Moreover, the pronounced enrichment of thorium and potassium exceeds the internationally established averages [1,5,27].

**Figure 8:**
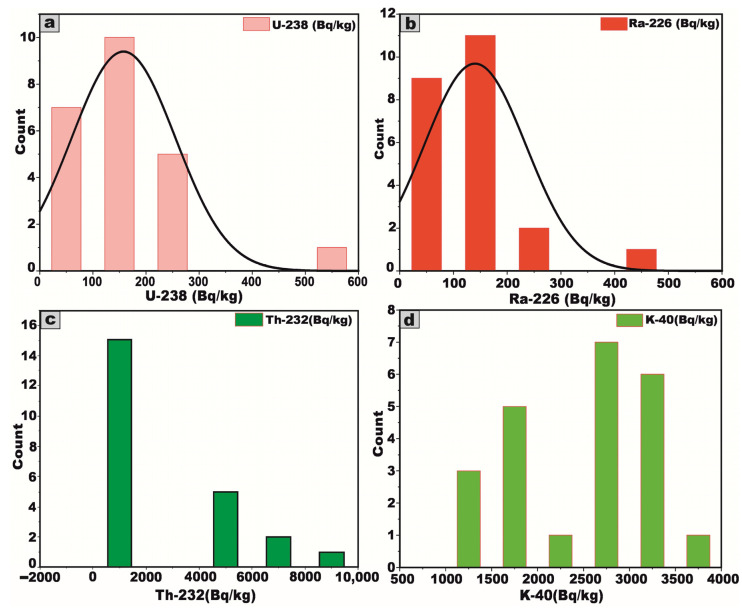
(**a**–**d**) Frequency distribution of radionuclide elements ^238^U, ^226^Ra, ^232^Th, and ^40^K (Bq/kg), respectively, for all studied granitic samples.

Skewness is a frequency distribution that refers to the degree of asymmetry observed around its central tendency. A distribution is considered skewed when it deviates from perfect symmetry, manifesting either a positive (right-tailed) or negative (left-tailed) inclination, thereby indicating an unequal dispersion of data values on either side of the mean. In Figure 8, the histograms illustrate the frequency distribution of specific activity concentration measurements (expressed in Bq/kg) for ^238^U, ^226^Ra, ^232^Th, and ^40^K across 23 distinct samples of the analyzed granitic rocks from the Arabian Shield, Saudi Arabia. The dataset exhibits that the skewness of the activity concentrations is positive in the current study, demonstrating the asymmetry of their distributions (Figure 8). The illustrated graph deviates from the canonical bell-shaped (normal) distribution, exhibiting a pronounced positive skewness as indicated by the predominance of higher-value observations extending toward the right tail (Figure 8).

**Figure 9:**
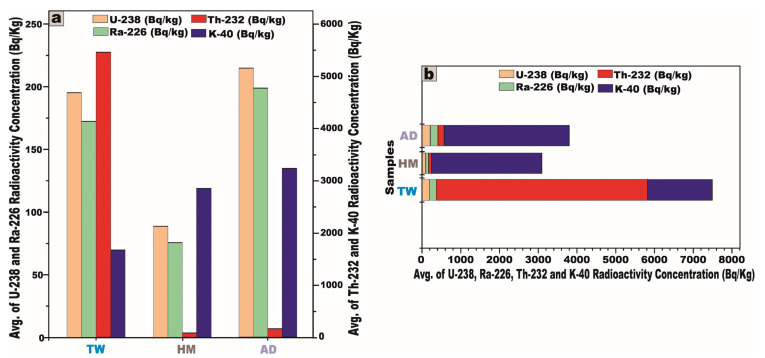
Comparison of the average specific activity concentrations of ^238^U, ^226^Ra, ^232^Th, and ^40^K in the granitic samples from the study area, Saudi Arabia, Arabian Shield. (**a**) Vertical column chart showing the average concentrations of each radionuclide individually; and (**b**) horizontal stacked bar chart illustrating the cumulative average contributions of each radionuclide to total radioactivity.

From the foregoing results, we noted that the mean activity concentrations of ^238^U, ^226^Ra, and ^40^K in the Abu Al Dod granitic stock (AD) are significantly higher than those observed in the Tawlah albite granite stock (TW) and Hamra alkali feldspar stock (HM) (Figure 9). This disparity can be ascribed to the K-metasomatic alteration process, which enhances potassium enrichment within the granitic stocks [28,29]. Furthermore, the elevated concentrations may be attributed to the geochemical enrichment of alkali feldspar minerals, particularly albite and potash feldspar, in tandem with the pervasive influence of alkali metasomatism, which leads to the mobilization and concentration of uranium and radium [13,30]. Additionally, the enhanced average activity concentration of ^232^Th in J. Tawlah albite granite (TW), relative to the concentrations observed in the Hamra alkali feldspar stock (HM) and the Abu Al Dod granitic pluton (AD) (Figure 9), can be ascribed to the effects of metasomatism and the overprints of greisenization facilitating the mobilization and re-deposition of thorium during the alteration of primary minerals [6,8].

**Figure 10:**
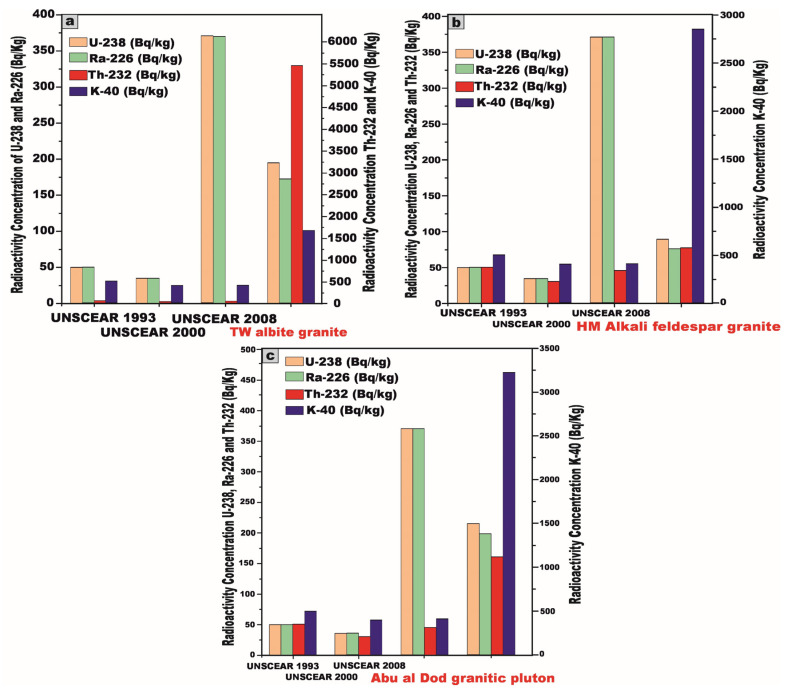
Comparison between specific activity concentrations ^238^U, ^226^Ra, ^232^Th, and ^40^K of granitic samples of our study and the world average concentrations of granitic rocks refers to (UNSCEAR, 1993, 2000, and 2008 [1,5,27]). (**a**) for Tawlah (TW) albite granite; (**b**) for Hamra (HM) alkali feldspar granite; and (**c**) for Abu Al Dod (AD) granitic stock. UNSCEAR, 1993 [1] values (^238^U, ^226^Ra = 50, ^232^Th = 50, and ^40^K = 500 Bq/kg), UNSCEAR, 2000 [5] values (^238^U, ^226^Ra = 35, ^232^Th = 30, and ^40^K = 400 Bq/kg); and UNSCEAR, 2008 [27] values (^238^U, ^226^Ra = 370, ^232^Th = 45, and ^40^K = 412 Bq/kg).

To facilitate a robust comparative evaluation of the mean specific activity concentrations of radionuclides in the investigated granitic rocks with globally reported reference values (Figure 10), we referenced UNSCEAR data from 1993 (^238^U, ^226^Ra = 50; ^232^Th = 50; and ^40^K = 500 Bq/kg), 2000 (^238^U, ^226^Ra = 35; ^232^Th = 30; and ^40^K = 400 Bq/kg), and 2008 (^238^U, ^226^Ra = 370; ^232^Th = 45; and ^40^K = 412 Bq/kg), revealing that although the measured activity concentrations of ^238^U (195.3, 88.66, and 214.3 Bq/kg) and ^226^Ra (172.4, 75.62, and 198.4 Bq/kg) in the TW, HM, and AD samples, respectively, were below the upper bounds of the UNSCEAR 2008 values [27], they still exceeded the lower baseline limits reported in the 1993 and 2000 editions [1,5], whereas the concentrations of ^232^Th (5453.8, 77.16, and 160.2 Bq/kg) and ^40^K (1670, 2846.2, and 3225 Bq/kg) in the corresponding samples were markedly elevated relative to all referenced UNSCEAR (1993, 2000, and 2008) global averages [1,5,27] (Figure 10).

The activity concentrations of ^238^U and ^226^Ra exhibit significant increases in the TW, HM, and AD samples, rising by [291%, 77%, and 329%] and [245%, 51%, and 297%], respectively, relative to the UNCEAR 1993 [1] averages (50 Bq/kg), with these increments intensifying to [458%, 153%, and 512%] and [393%, 116%, and 467%], respectively, when compared to the UNCEAR 2000 [5] benchmarks (35 Bq/kg).

The activity concentration of ^232^Th demonstrates extraordinary escalation, increasing by 10,807%, 54%, and 220% for TW, HM, and AD samples, respectively, relative to the UNCEAR 1993 [1] global average of 50 Bq/kg. Compared to the UNCEAR 2000 [5] baseline of 30 Bq/kg, these increments surge dramatically to 18,079%, 157%, and 434%. Against the UNCEAR 2008 [27] standard of 45 Bq/kg, the increases remain pronounced at 12,019%, 72%, and 256%, respectively.

Similarly, the activity concentration of ^40^K exhibits significant augmentation, rising by 234%, 469%, and 545% for TW, HM, and AD samples relative to the UNCEAR 1993 [1] global average of 500 Bq/kg. When measured against the UNCEAR 2000 [5] average of 400 Bq/kg, the increments intensify to 318%, 612%, and 706% while, relative to the UNCEAR 2008 [27] benchmark of 412 Bq/kg, the increases are 305%, 590%, and 683%, respectively.

### 5.2. Variation Diagrams

**Figure 11:**
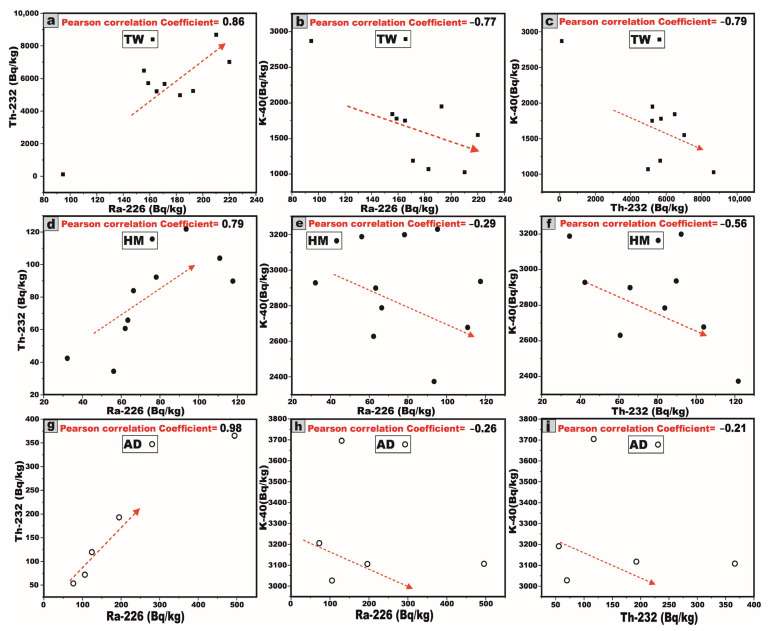
Variation diagrams between radionuclide elements ^226^Ra, ^232^Th, and ^40^K (Bq/kg) for the studied granitic samples: (**a**–**c**) for Tawlah Stock albite granites (TW), (**d**–**f**) for Hamra stock Alkali feldspar granites (HM), and (**g**–**i**) for Abu Al Dod granitic pluton (AD), Saudi Arabia, Arabian Shield. The red lines are the best-fit trendlines.

This analytical approach provides a robust statistical framework for assessing the degree of linear association between the studied radionuclides. A pronounced strong positive correlation is distinctly observed between ^226^Ra and ^232^Th within J. Tawlah albite granite (TW), Hamra alkali feldspar granite (HM), and J. Abu Al Dod granitic stock (AD) (Figure 11a,d,g). This strong linear relationship underscores the magmatic origin of the radionuclides ^226^Ra and ^232^Th [8,31,32], as well as the coexistence of these radionuclide elements in radioactive accessory minerals such as zircon, xenotime, and thorite.

For J. Tawlah albite granite (TW), a strong negative correlation is distinctly observed between ^226^Ra and ^232^Th with ^40^K, respectively (Figure 11b,c). In the case of J. Hamra alkali feldspar granite (HM), the correlations between ^226^Ra and ^232^Th with ^40^K are weak and moderately negative, respectively (Figure 11e,f). Within the G. Abu Al Dod granitic stock (AD), the relationships between ^226^Ra and ^232^Th with ^40^K exhibit an overall weak negative correlation (Figure 11h,i). These negative correlations suggest that these relationships to be potentially governed by post-magmatic alterations and the heterogeneous redistribution of ^226^Ra and ^232^Th enrichment.

These inter-relationships were rigorously substantiated through the computation of the Pearson correlation coefficient and the graphical representation of the best-fit regression line, meticulously generated using Origin software 2025 (Learning Edition).

The average ^232^Th/^226^Ra (^238^U) activity ratio in the investigated granitic samples reveals notable variability among the three plutons (Table 1). For TW albite granite, it exhibits a significantly elevated average ratio of 30.1, which far exceeds the typical crustal Th/Ra ratio (3.5) [33,34], suggesting considerable thorium enrichment and/or potential uranium leaching. In contrast, the HM alkali feldspar granite shows an average ratio of 1.04, while the AD granitic pluton displays a slightly lower average of 0.82. These discrepancies may reflect differing degrees of magmatic differentiation, post-magmatic hydrothermal alteration, and the enrichment of uranium within the host rocks.

### 5.3. Radiometric Hazards Assessment

#### 5.3.1. Radium Equivalent Activity (${Ra}_{eq}$)

For nearly four decades, radium equivalent activity (${Ra}_{eq}$) has served as a standardized parameter to enable the comparative assessment of radionuclide-specific activities in materials exhibiting varying concentrations of ^238^U, ^226^Ra, ^232^Th, and ^40^K. This metric represents a weighted summation of the activity concentrations of these radionuclides, based on the assumption that 10 Bq/kg of ^226^Ra, 7 Bq/kg of ^232^Th, and 130 Bq/kg of ^40^K contribute equivalently to external gamma radiation dose rates. Originally formulated by [35], this methodology provides a consistent and reliable framework for evaluating and comparing the radiological hazards associated with different geological and industrial materials, based on their radioactive content.

$$\;\;\;\;{Ra}_{eq}=C_{Ra}+\frac{{10C}_{Th}}{7}+\frac{{10C}_{K}}{130}\leq 370$$

where $C_{Ra}$,$\;C_{Th}$, and $C_{K}$ represent the specific activity concentrations of ^226^Ra, ^232^Th, and ^40^K, respectively. It is paramount to ensure that the radium equivalent concentration (${Ra}_{eq}$) remains below 370 Bq/kg to comply with radiological safety standards, thereby mitigating potential radiation exposure risks associated with material utilization.

**Figure 12:**
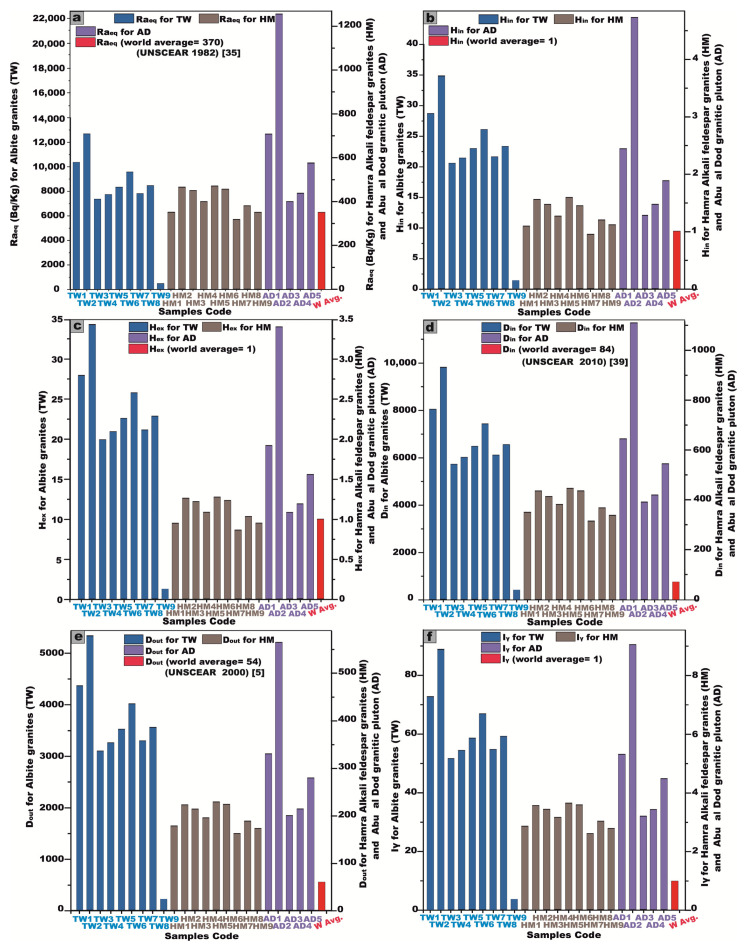

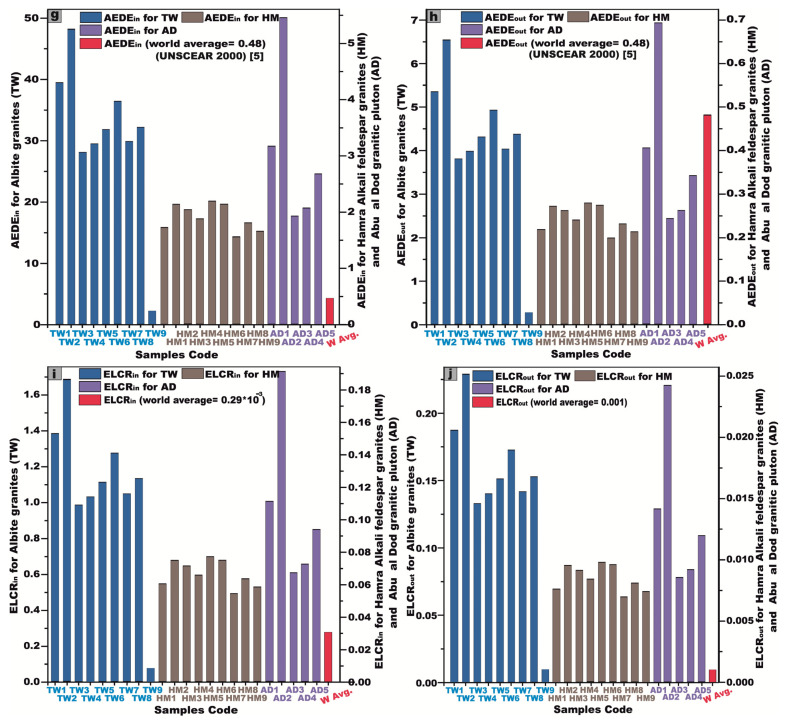
Comparison between radiological hazard indexes values ((**a**): Ra_eq_, (**b**): H_in_, (**c**): H_ex_, (**d**): D_in_, (**e**): D_out_, (**f**): I_γ_, (**g**): AEDE_in_, (**h**): AEDE_out_, (**i**): ELCR_in_, and (**j**): ELCR_out_) for the studied granitic samples and worldwide values. Symbols: TW = Tawlah Stock albite granites, HM = Hamra stock Alkali feldspar granites, and AD = Abu al Dod granitic pluton.

The ${Ra}_{eq}$ values for the Tawlah albite granite exhibit a wide range, fluctuating between 474.2 and 12,685.4 Bq/kg, with an elevated mean value of 8091.9 Bq/kg. Similarly, the Hamra alkali feldspar granite demonstrates ${Ra}_{eq}$ values spanning 317.9 to 471.6 Bq/kg, yielding an average of 404.8 Bq/kg. Meanwhile, the Abu Al Dod granitic pluton showcases values ranging from 399.3 to 1256.1 Bq/kg, with a mean of 675.3 Bq/kg (Table 2 and Figure 12a). Regrettably, all of these values exceed the recommended safety threshold of 370 Bq/kg (Figure 12a), underscoring significant radiological implications associated with their utilization, rendering them unsuitable for application as construction materials or interior decorative elements within residential environments.

#### 5.3.2. Internal and External Hazard Indices ($H_{in}$ and $H_{ex}$)

The internal hazard index ($H_{in}$) serves as a critical parameter for assessing the radiological risk associated with internal exposure to radon (^222^Rn) and its progeny, given that radon and its short-lived decay products pose significant health hazards, particularly to the respiratory system. This index is computed using the following equation:

$$H_{in}=\frac{C_{Ra}}{185}+\frac{C_{Th}}{259}+\frac{C_{K}}{4810}\leq 1$$

where $C_{Ra}$, $C_{Th}$, and $C_{K}$ are the activity concentrations of ^226^Ra, ^232^Th, and ^40^K, respectively.

The external hazards index ($H_{ex}$) represents a fundamental radiological parameter utilized in quantifying exposure factors for assessing natural gamma radiation hazards. This index serves as a critical metric for evaluating the potential radiological risks associated with external gamma exposure in various environmental settings and geological materials. As a standardized safety criterion, $H_{ex}$ is formulated based on the methodology proposed by Beretka and Mathew (1985) [36] and is mathematically expressed as follows:

$${\;\;\;\;\;H}_{ex}=\frac{C_{Ra}}{370}+\frac{C_{Th}}{259}+\frac{C_{K}}{4810}\leq 1$$

To ensure that radiation hazards remain at negligible and acceptable levels, both $H_{in}$ and $H_{ex}$ must not exceed the threshold value of 1. For the Tawlah albite granite,$\;H_{in}$ values range from 1.5 to 34.9 Bq/kg, with a mean of 22.3 Bq/kg, while $H_{ex}$ fluctuates between 1.3 and 34.3 Bq/kg, averaging 21.9 Bq/kg. In the Hamra alkali feldspar granite, $H_{in}$ spans from 0.9 to 1.6 Bq/kg, with an average of 1.3 Bq/kg, whereas $H_{ex}$ ranges from 0.9 to 1.3 Bq/kg, with a mean value of 1.1 Bq/kg. For the Abu Al Dod granitic pluton, $H_{in}$ varies between 1.3 and 4.7 Bq/kg, with an average of 2.4 Bq/kg, while $H_{ex}$ ranges from 1.1 to 3.4 Bq/kg, averaging 1.8 Bq/kg (Table 2 and Figure 12b,c). Regrettably, the calculated $H_{in}$ and $H_{ex}$ values exceed the recommended safety threshold ($H_{in}$ and $H_{ex}$ ≤ 1) [37], underscoring potential radiological concerns that warrant further scrutiny (Table 2 and Figure 12b,c).

#### 5.3.3. Gamma Absorbed Dose Rate in Air (D)

The gamma absorbed dose rate in air is the gamma dose at 1 m above the ground level [38].

$$D_{in}(\mathrm{nGy}/h)=(0.92C_{Ra})+(1.1C_{Th})+(0.082C_{K})$$

$$D_{out}\;(\mathrm{nGy}/h)=(0.436C_{Ra})+(0.599C_{Th})+(0.0417C_{K})$$

where $D_{in}$ is the indoor absorbed gamma dose rate and $D_{out}$ is the outdoor absorbed gamma dose rate.

It is well established that the global average value for the indoor absorbed gamma dose rate ($D_{in}$) under typical granitic conditions is 84 nGy/h, as stipulated by [5,39]. Moreover, the worldwide mean value for the outdoor gamma dose rate ($D_{out}$), corresponding to standard soil conditions, has been documented at 54 nGy/h, under UNSCEAR 2000 [5].

In the present study, the recorded $D_{in}$ for the Tawlah albite granite pluton exhibits a substantial range, spanning from 444.7 to 9822.6 nGy/h, with an elevated mean value of 6294.7 nGy/h. Correspondingly, the $D_{out}$ to the same stock fluctuates between 227.5 and 5332.2 nGy/h, yielding an average of 3411.6 nGy/h (Table 2 and Figure 12d,e). For G. Hamra alkali feldspar granite, $D_{in}$ values range from 316.3 to 447.6 nGy/h, with an average of 387.8 nGy/h, while $\;D_{out}$ spans from 161.5 to 227.4 nGy/h, averaging 197.9 nGy/h. In the case of the Abu Al Dod granitic pluton, $D_{in}$ ranges from 391.2 to 1111.8 nGy/h, with a mean value of 623.2 nGy/h, whereas $D_{out}$ fluctuates between 198.8 and 564.2 nGy/h, yielding an average of 316.9 nGy/h (Table 2 and Figure 12d,e). Notably, these measured dose rates exceed the globally recognized safety benchmarks.

#### 5.3.4. Representative Gamma Index (I_γ_)

The representative level index (I_γ_) functions as a quantitative metric for evaluating the γ-radiation hazard associated with the presence of naturally occurring radionuclides in the analyzed samples, as stipulated by OECD, 1979 [40]. It can be calculated as follows:

I_γ_ = C_Ra_/150 + C_Th_/100 + C_k_/1500

The gamma index (I_γ_) serves a dual purpose, functioning as both a quantitative assessor of γ-radiation hazards in the examined samples and a predictive indicator of the annual dose rate stemming from the excessive external gamma radiation emitted by surface materials. As a critical radiological screening parameter, it plays an indispensable role in evaluating materials that might pose potential health risks, particularly when employed in construction and infrastructure development [41].

The statistical analyses presented in Table 2 and Figure 12f illustrate the gamma index (I_γ_) values across the studied granitic formations. Specifically for Tawlah Stock albite granites, I_γ_ values range from 3.7 to 88.9 mSv/y, with an elevated mean of 56.8 mSv/y. In the case of Hamra Stock alkali feldspar granites, values vary from 2.6 to 3.7 mSv/y, averaging 3.17 mSv/y. Similarly, for the Abu Al Dod granitic pluton, I_γ_ spans from 3.2 to 9.1 mSv/y, with a mean value of 5.1 mSv/y. Notably, these recorded values significantly surpass the globally recommended threshold of 1, indicating a heightened radiological concern.

#### 5.3.5. Annual Effective Dose Rate (AEDE)

The annual indoor and outdoor effective dose equivalents (AEDE_in_ and AEDE_out_) are calculated as follows:

$${\mathrm{AEDE}}_{\mathrm{in}}\;(\mathrm{mSv}/\mathrm{year})\;=\;D_{\mathrm{in}}\;(\mathrm{nGy}/h)\;\times \;8760\;(h/\mathrm{year})\;\times \;0.8\;\times \;0.7\;(\mathrm{Sv}/\mathrm{Gy})\;\times \;\frac{10^{3}mSv}{10^{9}nGy}$$

$${\mathrm{AEDE}}_{\mathrm{out}}(\mathrm{mSv}/\mathrm{year})=D_{\mathrm{out}}\;(\mathrm{nGy}/h)\;\times \;8760\;(h/\mathrm{year})\;\times \;0.2\;\times \;0.7\;(\mathrm{Sv}/\mathrm{Gy})\;\times \;\frac{10^{3}mSv}{10^{9}nGy}$$

The conversion coefficient for translating the absorbed dose in air to the effective dose for adults has been established at 0.7 Sv/Gy. Furthermore, occupancy factors of 0.8 for indoor environments and 0.2 for outdoor exposure were employed according to UNSCEAR 1993 [1]. The global average annual effective dose equivalent (AEDE) resulting from terrestrial gamma radiation, whether indoors or outdoors, is reported to be 0.48 mSv/year, as stipulated by UNSCEAR 2000 [5].

In the present study, the AEDE_in_ exhibited a substantial variation across different granitic formations. For the Tawlah albite granite, AEDE_in_ ranged from 2.2 to 48.2 Sv/Gy, with an elevated mean value of 30.9 Sv/Gy. In contrast, the Hamra alkali feldspar granite stock (HM) demonstrated AEDE_in_ values spanning from 1.6 to 2.2 Sv/Gy, averaging 1.9 Sv/Gy. Similarly, for the Abu Al Dod granitic pluton (AD), AEDE_in_ varied between 1.9 and 5.4 Sv/Gy, with a mean value of 3.1 Sv/Gy. Notably, the computed AEDE_in_ values exceed the internationally established safety threshold (Table 2 and Figure 12g).

In the current investigation, the AEDE_out_ exhibited notable variability across the studied granitic stocks. For the Tawlah albite granite, the AEDE_out_ values ranged from 0.3 to 6.5 Sv/Gy, with an elevated mean value of 4.2 Sv/Gy. Conversely, J. Hamra alkali feldspar granite exhibited AEDE_out_ values spanning from 0.19 to 0.28 Sv/Gy, with a modest mean of 0.24 Sv/Gy. Likewise, for the Abu Al Dod granitic stock, AEDE_out_ values fluctuated between 0.24 and 0.7 Sv/Gy, with an average of 0.4 Sv/Gy (Table 2 and Figure 12h). Notably, the AEDE_out_ levels for Tawlah albite granite stock surpass the internationally recognized safety threshold (Table 2 and Figure 12h), while the values observed for the Hamra stock and Abu Al Dod stock remain within the permissible limits.

#### 5.3.6. Excess Lifetime Cancer Risk (ELCR)

With the help of AEDE values, the excess lifetime cancer risk (ELCR) is calculated as follows:

ELCR_in_ (×10^−3^) = AEDE_in_ × Average duration of life (DL) × Risk factor (RF)

ELCRout (×10^−3^) = AEDE_out_ × Average duration of life (DL) × Risk factor (RF)

where AEDE is the annual effective dose rate equivalent, DL is the life expectancy (70 years), and RF is the fatal risk factor (0.5 SV^−1^), i.e., the fatal cancer risk per Sievert is 0.05 for the public [42,43].

For the Tawlah albite granites, the estimated ELCR_in_ exhibits a substantial variation, ranging from 0.08 to 1.7, with an elevated mean value of 1.08. The Hamra alkali feldspar granites display a relatively lower ELCR_in_, fluctuating between 0.05 and 0.08, with an average of 0.07. Similarly, for the Abu Al Dod granitic stock, ELCR_in_ spans from 0.07 to 0.19, with an average value of 0.11 (Table 2 and Figure 12i).

The ELCR_out_ values for Tawlah Stock albite granites range between 0.01 and 0.23, averaging at 0.15. Meanwhile, the Hamra Stock alkali feldspar granites exhibit minimal ELCR_out_ values, remaining constant at 0.01. Likewise, for the Abu Al Dod granitic stock, the ELCR_out_ values range from 0.01 to 0.02, with an average of 0.01 (Table 2 and Figure 12j).

The values of ELCR_in_ and ELCR_out_ are higher than the world value (0.29 × 10^–3^ (=0.00029)) and (1.168 × 10^–3^ (=0.001)), respectively [44] (Table 2 and Figure 12i,j).

#### 5.3.7. Effective Dose Rate for Different Body Organs and Tissues (D_organ_)

This methodology ensures an accurate assessment of radiation exposure to individual organs, facilitating a comprehensive evaluation of potential radiological health risks. The organ-specific effective dose rate (D_organ_) can be precisely determined using the following equation [42,45]:

D_organ_ = AEDE × F

where D_organ_ is the effective dose rate to the organs, AEDE represents the annual effective dose equivalent, and F is the conversion factor of the organ dose from the air dose.

The quantification of the dose rates imparted to various organs, along with the corresponding average occupancy factors (F) for distinct tissues, is meticulously outlined in Table 3. This table provides a comprehensive analysis of the effective dose rates for specific organs, considering their respective occupancy factors. Based on the empirical data and results delineated in Table 3, it is evident that the radiation doses received by the investigated organs from granitic rock exposures surpass the internationally established tolerable threshold for the annual organ dose intake (D_organ_ = AEDE × F), which is set at 1.0 mSv [46].

**Figure 13:**
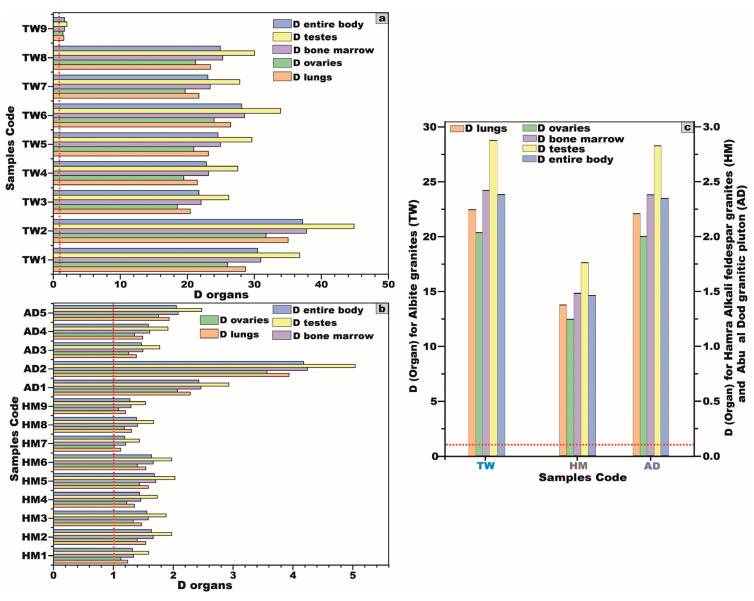
(**a**,**b**) Bar diagram to compare between dose rates (D) of the different organs for TW and HM samples, respectively, and (**c**) comparison of the average dose rate (D) of the different organs. Symbols: TW = Tawlah Stock albite granites, HM = Hamra stock Alkali feldspar granites, AD = Abu al Dod granitic pluton, and red line = international limit for annual organ dose intake.

A striking observation emerges from the data: the testes exhibit the highest radiation susceptibility, with mean values of 28.8, 1.8, and 2.8 for Tawlah Stock albite granites, Hamra Stock alkali feldspar granites, and the Abu Al Dod granitic stock, respectively. In contrast, ovaries display the lowest radiation sensitivity, with corresponding mean values of 20.3, 1.2, and 1.9 for the same geological formations, respectively. Consequently, these findings suggest that males are inherently more vulnerable to radiation exposure than females (Figure 13).

**Figure 14:**
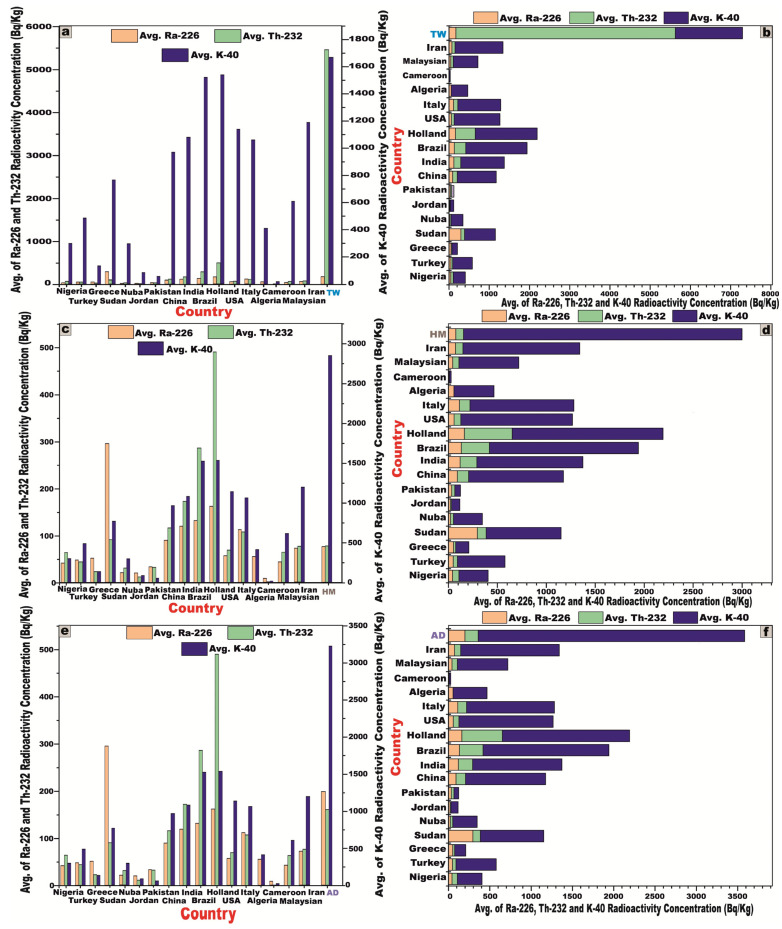
Comparison between ^226^Ra, ^232^Th, and ^40^K Bq/kg of some granitic rocks (for building materials) in countries around the world and our study using column diagram and stacked bar diagram: (**a**,**b**) for Tawlah Stock albite granites (TW), (**c**,**d**) for Hamra stock Alkali feldspar granites (HM), and (**e**,**f**) for Abu Al Dod granitic pluton (AD).

**Figure 15:**
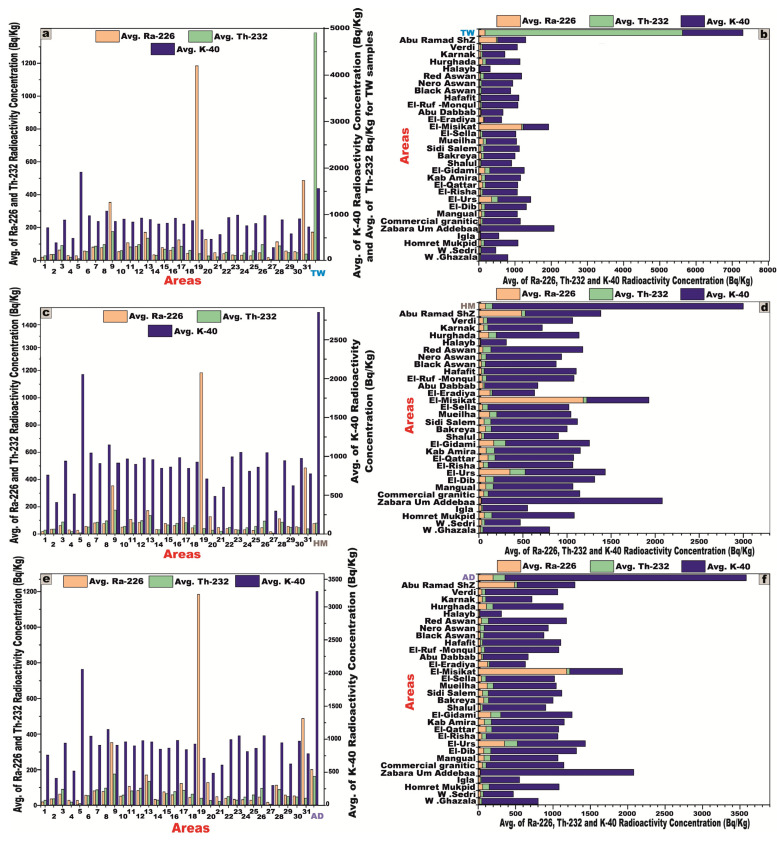
Comparison between ^226^Ra, ^232^Th, and ^40^K Bq/kg of some granitic rocks (for building materials) in Egypt and our study using column diagram and using stacked bar diagram: (**a**,**b**) for Tawlah Stock albite granites (TW), (**c**,**d**) for Hamra stock Alkali feldspar granites (HM), and (**e**,**f**) for Abu Al Dod granitic pluton (AD).

A comprehensive comparative analysis was carried out between the granitic samples from the studied area and those documented in various regions across the world (Table 4), employing column and stacked bar charts to facilitate a visual interpretation of the data (Figure 14). Furthermore, a regional-scale comparison was executed between the investigated granites and their counterparts from the Sinai Peninsula and the Eastern Desert (Table 5), using the same graphical methodologies to highlight disparities in natural radioelement concentrations (Figure 15).
1W. Ghazala8El-Dib15Bakreya22El-Ruf-Monqul29Karnak2W. Sedri9El-Urs16Sidi Salem23Hafafit30Verdi3Homret Mukpid10El-Risha17Mueilha24Black Aswan31Abu Ramad Shz4Igla11El-Qattar18El-Sella25Nero Aswan
TW5Zabara-Um Addebaa belt12Kab Amira19El-Misikat26Red AswanHM6Commercial granitic (7 types)13El-Gidami20El-Eradiya27HalaybAD7Mangual14Shalul21Abu Dabbab28Hurghada


**Figure 16:**
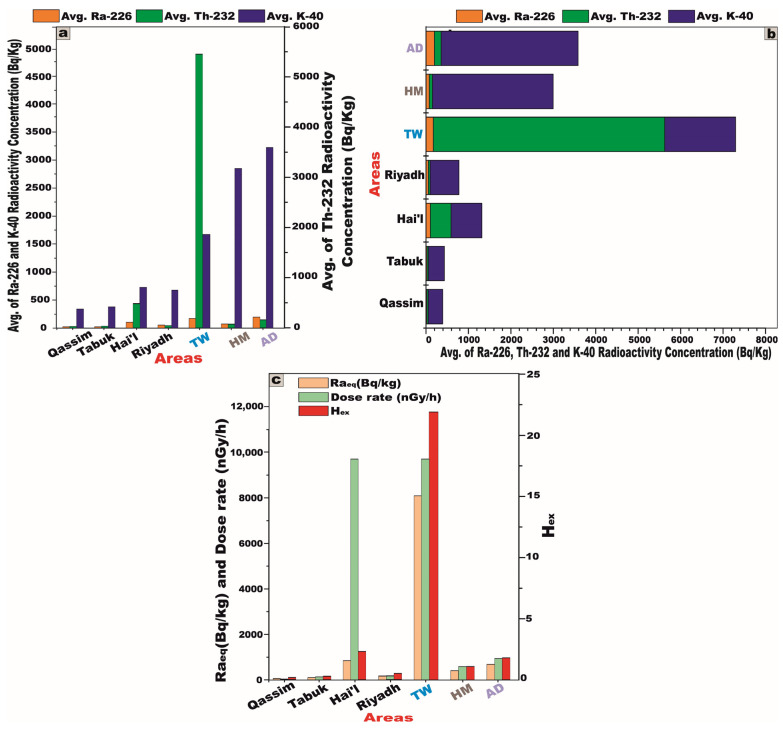
Comparison between ^226^Ra, ^232^Th, and ^40^K Bq/kg of some granitic rocks (for building materials) in Saudi Arabia and our study areas, using (**a**) column diagram, (**b**) stacked bar diagram, and (**c**) comparison between Ra_eq_ (Bq/kg), D (nGy/h), and H_ex_ of some granitic rocks (for building materials) in Saudi Arabia and our study areas, using column diagram. TW = Tawlah Stock albite granites, HM = Hamra stock Alkali feldspar granites, and AD = Abu Al Dod granitic pluton.

A comparative analysis was conducted between the studied granitic samples and selected granitic plutons from various regions in Saudi Arabia (Table 6), using column and stacked bar diagrams (Figure 16a,b). The key of some of the radiological parameters, such as Ra_eq_, the absorbed dose rate (D), and the external hazard index (H_ex_), were also compared for granites commonly used as building materials in the same regions within Saudi Arabia and those from the current study area (Figure 16c).

### 5.4. Geochemical Characteristics

#### 5.4.1. Distribution of Ra (U), Th, and K

It is a well-established fact that the activity concentrations of ^238^U, ^226^Ra, ^232^Th, and ^40^K, as determined through radiometric methodologies, exhibit equivalence to the concentrations of U (Ra), Th, and K obtained via chemical analytical techniques [6,8,27].

**Figure 17:**
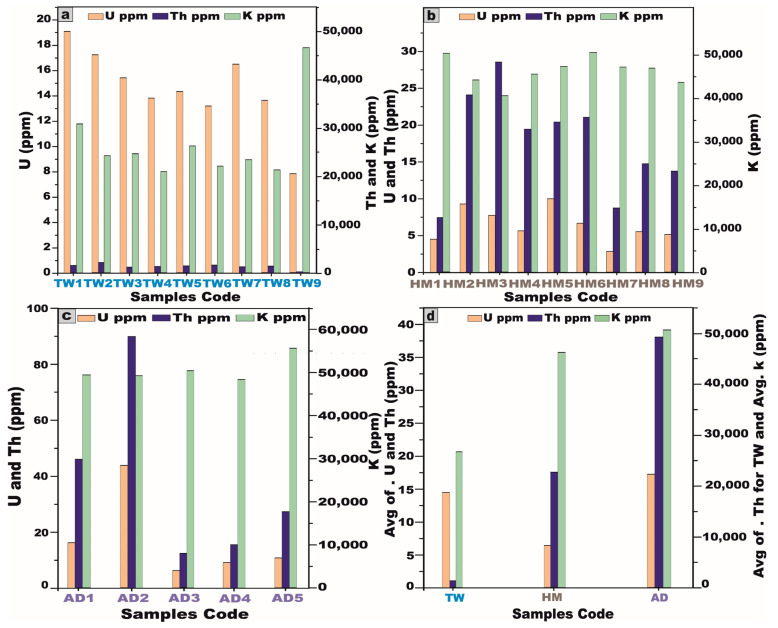
Plot of the elemental concentrations of radionuclide elements U, Th, and K (ppm) relevant to chemical analysis: (**a**) for Tawlah Stock albite granite (TW), (**b**) for Hamra stock Alkali feldspar granites (HM),(**c**) for Abu Al Dod granitic pluton (AD), and (**d**) Plot of the elemental concentrations to the average of radionuclide elements U, Th, and K (ppm) relevant to chemical analysis to all granitic samples, Saudi Arabia, Arabian Shield.

A comprehensive geochemical analysis was conducted on 14 representative samples from the main rock types of J. Hamra and J. Abu Al Dod to determine their U, Th, and K contents. These analyses were performed at the Specialized Analysis (GSSA) laboratories in Jeddah, Saudi Arabia, utilizing X-ray fluorescence (XRF) spectrometry (Table 7). In contrast, the geochemical data for an additional nine samples from J. Tawlah, targeting the same elemental suite, were sourced from the published dataset of [11] (Table 7 and Figure 17).

The elemental concentrations of U (Ra) exhibit notable variations across the investigated granitic rock units. Specifically, in Tawlah albite granites, U (Ra) values range from 7.8 to 19.1 ppm, with an average concentration of 14.5 ppm. In Hamra alkali feldspar granites, the concentrations span from 2.8 to 10.0 ppm, averaging 6.4 ppm. Meanwhile, in Abu Al Dod granitic pluton, U (Ra) values fluctuate between 6.2 and 43.7 ppm, with a mean concentration of 17.2 ppm (Table 7 and Figure 17).

The Th concentrations exhibit substantial variability among the analyzed granitic rock units. In Tawlah albite granites, Th concentrations range from 25.5 to 2124.8 ppm, with an average value of 1317.9 ppm, reflecting significant enrichment. Conversely, in Hamra alkali feldspar granites, Th concentrations display a narrower range, spanning 7.4 to 28.5 ppm, with a mean concentration of 17.5 ppm. Meanwhile, in Abu Al Dod granitic stock, the values fluctuate between 12.2 and 89.8 ppm, averaging 38.1 ppm (Table 7 and Figure 17).

In Tawlah albite granites, K concentrations range from 20,900 to 46,500 ppm, with an average value of 26,688.9 ppm, indicating relatively low enrichment. In contrast, Hamra alkali feldspar granites display a slightly elevated range, spanning 40,500 to 50,500 ppm, with a mean concentration of 46,211 ppm. Meanwhile, in Abu Al Dod granitic stock, K values fluctuate between 48,400 and 55,600 ppm, averaging 50,620 ppm (Table 7 and Figure 17). Please be sure to note the values of K for all stocks.

Among the investigated plutonic bodies, the Abu Al Dod granitic stock exhibits the highest average concentrations of U and K, with mean values of approximately 17.2 ppm and 50,620 ppm, respectively. In contrast, the Tawlah albite granites are distinguished by their exceptionally elevated Th content, registering an average concentration of 1317.9 ppm (Table 7 and Figure 17).

**Figure 18:**
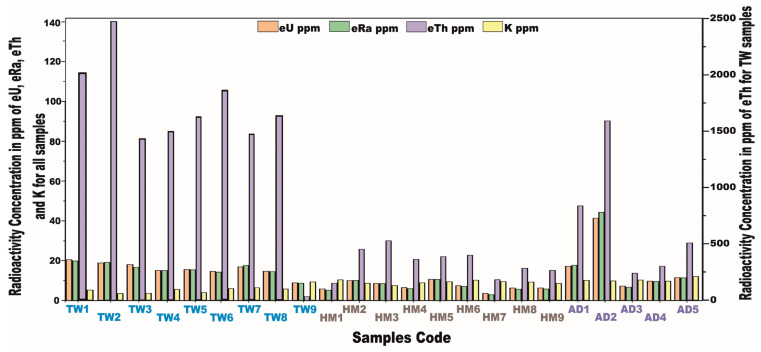
Plot of the radioactivity concentrations of radionuclide elements eU, eRa, eTh, and K (ppm) due to the transformation from Bq/kg into ppm for Tawlah Stock albite granites (TW), Hamra stock Alkali feldspar granites (HM), and Abu Al Dod granitic pluton (AD), Saudi Arabia, Arabian Shield. eU: equivalent uranium, eTh: equivalent thorium, and eRa: equivalent radium, derived from gamma-ray spectrometry, assuming radioactive equilibrium.

The radiometric data were obtained in parts per million (ppm) following the conversion of the activity concentrations of radionuclide elements from Bq/kg to ppm, employing the methodological framework delineated by Malczewski et al., 2004 [74] (Table 8 and Figure 18).

The eU concentrations, expressed in ppm, exhibit a notable range across the investigated granitic rock units, Saudi Arabia, Arabian Shield. Specifically, in Tawlah albite granites, eU values fluctuate between 8.8 and 20.5 ppm, with an average concentration of 15.8 ppm. In Hamra alkali feldspar granites, the concentrations vary from 3.6 to 10.6 ppm, averaging 7.2 ppm. Meanwhile, in Abu Al Dod granitic stock, the concentrations span from 7.1 to 41.5 ppm, with a mean value of 17.4 ppm (Table 8).

Concerning the eRa concentrations in ppm across the granitic stocks, for Tawlah albite granites, the eRa values fluctuate between 8.5 and 19.8 ppm, with an average concentration of 15.5 ppm. Hamra alkali feldspar granites exhibit eRa concentrations ranging from 2.9 to 10.6 ppm, with a mean value of 6.8 ppm. Meanwhile, in the Abu Al Dod granitic stock, the eRa concentrations present a relatively broader range, spanning from 6.7 to 44.5 ppm, with an average of 17.9 ppm (Table 8).

For the eTh concentrations, the Tawlah albite granites display a concentration range significantly fluctuating between 27.4 and 2137.3 ppm, with an average of 1343.3 ppm, indicating pronounced enrichment. In Hamra alkali feldspar granites, eTh spans from 8.5 to 29.9 ppm, with a mean concentration of 19 ppm. Meanwhile, in the Abu Al Dod granitic stock, eTh concentrations range from 13.7 to 90.3 ppm, averaging 39.5 ppm (Table 8). Regarding the K ppm concentrations, Tawlah albite granites show values that fluctuate between 3.3 and 9.2 ppm, with an average concentration of 5.3 ppm. Related to the Hamra granites, the K concentrations span from 7.6 to 10.2 ppm, yielding a mean value of 9.1 ppm. Meanwhile, in Abu Al Dod granitic pluton, the K concentrations display a relatively narrower range, varying between 9.7 and 11.8 ppm, with an average of 10.3 ppm (Table 8).

**Figure 19:**
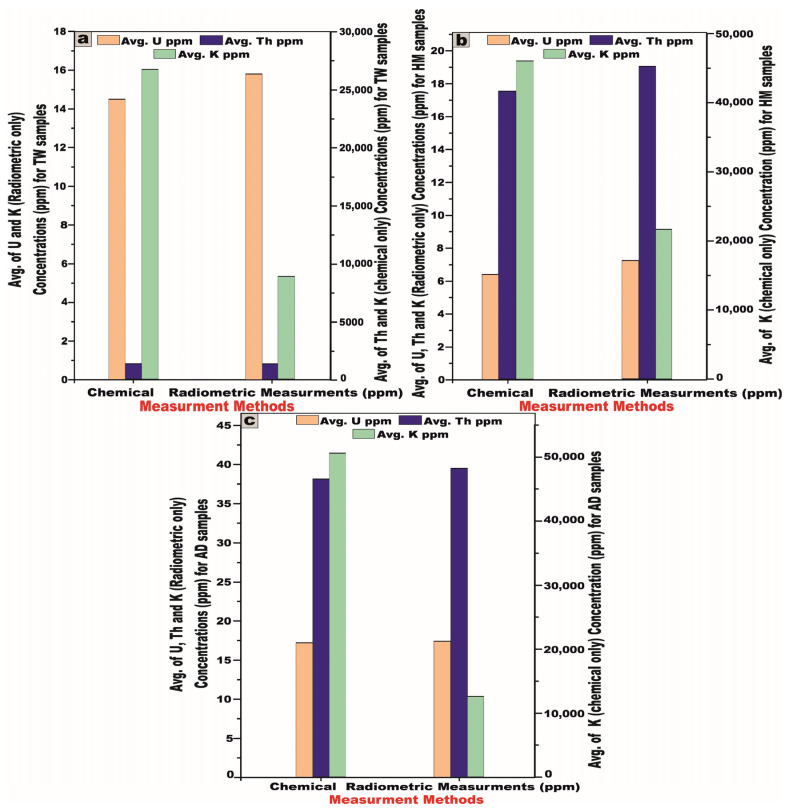
Comparison between the average of radionuclide elements (U (Ra), Th, and K) of the studied granites, using chemical analysis and radiometric spectroscopy measurements (NaI-Tl): (**a**) for Tawlah Stock albite granites (TW), (**b**) for Hamra Stock Alkali feldspar granites (HM), and (**c**) Abu Al Dod granitic pluton (AD).

By juxtaposing the radionuclide concentrations of U (Ra), Th, and K (expressed in ppm) obtained via chemical analysis (Table 7) with those quantified using the NaI (Tl) scintillation detectors (Table 8), the radiometric measurements consistently yield higher average concentrations of U (Ra) and Th compared to those determined through XRF analysis to all granitic samples, indicating uranium leaching (Figure 19). However, the K ppm concentrations obtained through a chemical analysis exhibit significantly higher values compared to those derived from radiometric measurements to all granitic samples, indicating K enrichment.

#### 5.4.2. Radiochemical Element Data Interpretation

The comprehensive radiochemical dataset encompassing U, Th, and K concentrations for the investigated granitic plutons within the Saudi Arabian Shield (Table 7) was meticulously acquired to elucidate the geochemical signatures and trace the genesis of these naturally occurring radioactive elements.

**Figure 20:**
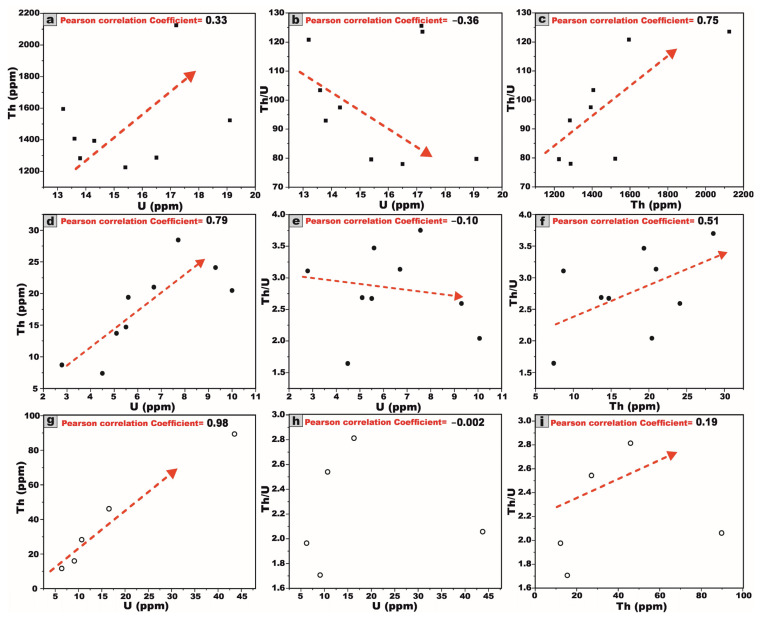
Variation diagrams between radionuclide elements (U and Th ppm) relevant to chemical analysis for the studied granitic samples: (**a**–**c**) for Tawlah Stock albite granites (TW), (**d**–**f**) for Hamra stock alkali feldspar granites (HM), and (**g**–**i**) Abu Al Dod granitic pluton (AD), Saudi Arabia, Arabian Shield. Symbols as in Figure 11. The red lines are the best-fit trendlines. Notes: chemical analysis of samples from J. Tawlah (TW) (after [11]).

Based on the provided chemical data of U and Th concentrations (ppm) for the studied granite stocks (Table 7 and Figure 20a–i), a discernible positive correlation is evident between U and Th, wherein the progressive increase in the U concentrations corresponds with a modest yet consistent elevation in Th levels (Figure 20a,d,g) across the TW granites, HM granites, and AD granitic pluton. This trend underscores the magmatic imprint characteristic of the early stages of crystallization, a phenomenon typically observed in felsic igneous bodies.

Conversely, a negative correlation is observed between the U concentration and the Th/U ratio, wherein an increase in the U content corresponds to a decline in the Th/U ratio for the TW granites, HM granites, and AD granitic stock (extremely weakly negative or potentially absent), respectively (Figure 20b,e,h). This trend signifies uranium enrichment relative to thorium depletion, likely attributable to hydrothermal alteration processes. Such an alteration facilitates the remobilization and redistribution of uranium, resulting in localized U enrichment while thorium remains largely immobile, thereby causing a reduction in the Th/U ratio.

A pronounced positive correlation between thorium Th and the Th/U ratio is observed for the TW and HM granites and the AD granitic pluton, respectively (Figure 20c,f,i). This trend strongly implies a preferential thorium enrichment relative to uranium, which may be attributed to magmatic differentiation.

**Figure 21:**
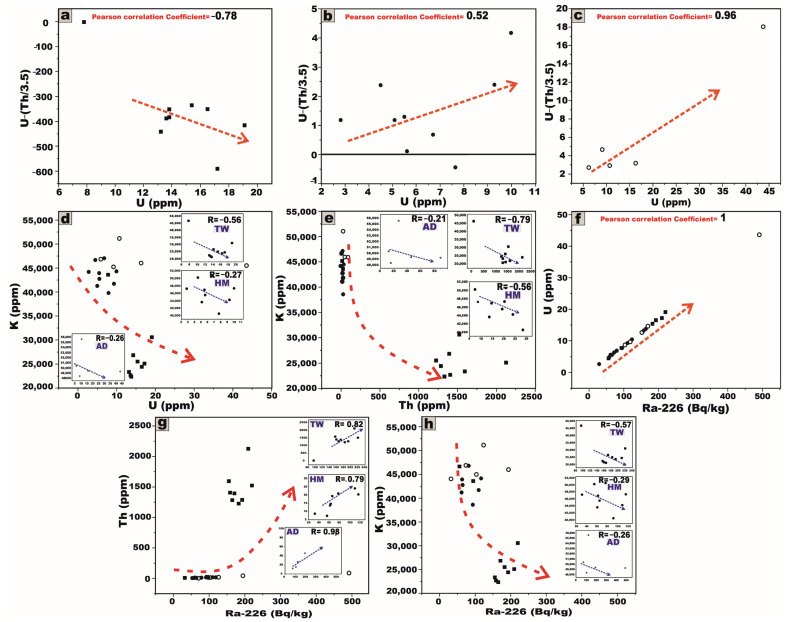
Variation diagrams between U and U−Th/3.5 (ppm) relevant to chemical analysis for the studied granitic samples: (**a**) for Tawlah Stock albite granites (TW), (**b**) for Hamra Stock alkali feldspar granites (HM), (**c**) Abu Al Dod granitic pluton (AD), (**d**) variation diagrams between U and K (ppm) for all granitic plutons, (**e**) between Th and K (ppm) for all granitic plutons, (**f**) between ^226^Ra (Bq/kg) and U (ppm) for all granitic plutons, (**g**) between ^226^Ra (Bq/kg) and Th (ppm) for all granitic plutons, and (**h**) between ^226^Ra (Bq/kg) and K (ppm) for all granitic plutons, Saudi Arabia, Arabian Shield. Symbols as in Figure 11. The red lines are the best-fit trendlines.

The correlation between U and (U–Th/3.5) (Figure 21a) for TW granites exhibits a pronounced negative trend. This strong inverse relationship signifies substantial uranium leaching (depletion). Conversely, the same correlation (Figure 21b,c) for HM granites and AD granitic pluton exhibits a pronounced moderate and strong positive trend, respectively. Those correlations are indicative of substantial uranium enrichment, likely driven by post-magmatic processes such as hydrothermal alterations [30,31,75,76].

A moderate and weak negative relationship is observed between U and K within the TW, HM, and AD granitic samples, respectively (Figure 21d). Likewise, Th and K show strong, moderate, and weak negative correlations in the TW, HM, and AD granitic samples, respectively (Figure 21e), likely reflecting the varying intensity of the alkali metasomatism affecting these granitic bodies.

Furthermore, the relationships between the radiometric ^226^Ra activity (Bq/kg) and the geochemically determined concentrations of U, Th, and K across all examined granitic plutons reveal strong positive correlations with U and Th through all the granitic samples (Figure 21f,g). In contrast, K exhibits moderate and weak negative correlations with ^226^Ra in the TW, HM, and AD granitic samples, respectively (Figure 21h).

All observed inter-relationships were statistically corroborated through factor analysis, employing Pearson correlation coefficients computed via the Origin software.

It is well established that the typical crustal Th/U ratio generally falls within the range of 2.5 to 5 [33,77]. The Th/U ratio exhibits marked variation across the investigated granitic bodies. In the Tawlah albite granites, the ratio ranges from 3.3 to 123.5, with a notably elevated average of 86.5, suggesting significant uranium depletion likely due to leaching processes (Table 7). In contrast, the Hamra alkali feldspar granites display a Th/U ratio lower than the average of the Earth’s crust ratio, ranging from 1.6 to 3.7 with a mean value of 2.8, reflecting uranium enrichment. The Abu Al Dod granitic pluton shows a ratio varying from 1.7 to 2.8 and averaging at 2.2 (Table 7), which may indicate uranium enrichment possibly linked to hydrothermal activity (e.g., [8,75]).

#### 5.4.3. Radioactive Element Data Interpretation

Table 8 and Figure 18 present the radiometric datasets detailing the concentrations of the radionuclides U, Ra, Th, and K ppm for the investigated granitic pluton, following their conversion from Bq/kg to ppm.

**Figure 22:**
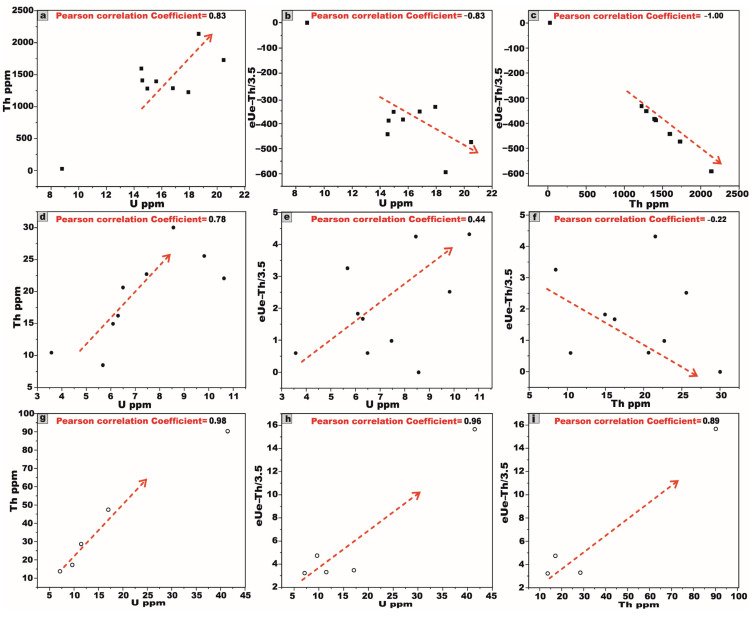
Variation diagrams between radionuclide elements after the conversion from Bq/kg to ppm values, between (U, Th), (U, eU–eTh/3.5), and (Th, eU–eTh/3.5), respectively: (**a**–**c**) for Tawlah Stock albite granites (TW), (**d**–**f**) for Hamra Stock Alkali feldspar granites (HM), and (**g**–**i**) for Abu al Dod granitic pluton (AD), Saudi Arabia, Arabian Shield. Symbols as in Figure 11. The red lines are the best-fit trendlines.

The correlation between eU and eTh demonstrates a moderately strong positive association across all granitic samples from the TW, HM, and AD stocks (Figure 22a,d,g). This relationship substantiates the magmatic origin of the radionuclides and is likely attributable to the presence of thorite and related accessory minerals, as corroborated by petrographic investigations.

Furthermore, the relationship between eU and eU–eTh/3.5 exhibits a pronounced negative correlation in the TW granite (Figure 22b), indicating significant uranium leaching. In contrast, HM and AD granitic plutons display moderate and strong positive correlations, respectively (Figure 22e,h), suggestive of uranium enrichment and mobilization processes by hydrothermal fluid activity. This interpretation is further supported by the elevated average value of the (eU–eTh/3.5), calculated at 1.7 > 0.

The correlation between eTh and eU–eTh/3.5 exhibits a strong and weak negative trend in TW and HM granitic plutons, respectively, indicating Th dominance and relative uranium depletion (Figure 22c,f). Conversely, AD pluton demonstrates a strong positive correlation (Figure 22i), suggestive of significant uranium enrichment, as the value of the (eU–eTh/3.5) calculated at 6.1 > 0.

The average radiometric eTh/eU ratio for TW granite is approximately 80.9 ppm (Table 8), which significantly exceeds the average continental crustal value of ~3.8 [78]. This strongly suggests substantial uranium leaching. In contrast, HM and AD granitic stocks exhibit notably lower eTh/eU ratios of 2.6 and 2.2 ppm, respectively (Table 8), both of which fall below the expected crustal benchmark. Such reduced ratios are indicative of considerable uranium enrichment [75].

#### 5.4.4. Equilibrium/Disequilibrium State (D-Factor)

The assessment of uranium equilibrium and disequilibrium states was first introduced by Hansink, 1976 [79], who defined the D-factor as the ratio between chemically determined uranium concentrations (Uc) and those obtained through radiometric measurements (Ur). An equilibrium state is indicated when this ratio approaches unity (D = 1). Conversely, any positive or negative deviation from unity signifies a disequilibrium condition, implying that uranium has either been mobilized or leached since its original emplacement. In the examined granitic stocks, the average Uc values were measured at 14.5, 6.4, and 17.2 ppm for TW, HM, and AD stocks, respectively. Correspondingly, the Ur were recorded at 15.8, 7.2, and 17.4 for the same units (Table 7). The resulting Uc/Ur ratios, 0.92 for TW, 0.87 for HM, and 0.95 for AD, are all below unity, unequivocally indicating the disequilibrium state. This deviation suggests the post-magmatic leaching or redistribution of uranium, likely driven by secondary alteration processes that disrupted the original isotopic balance [9].

## 6. Remote Sensing Data Interpretation

Landsat-9 satellite imagery was utilized as an effective tool to enhance the lithological discrimination and cartographic delineation of the granitic rock units within the investigated stocks [80]. Furthermore, it proved instrumental in the detection and spatial characterization of alteration zones associated with radioactive mineralizations, associated with the younger granites, across parts of the Arabian Shield in Saudi Arabia.

**Figure 23:**
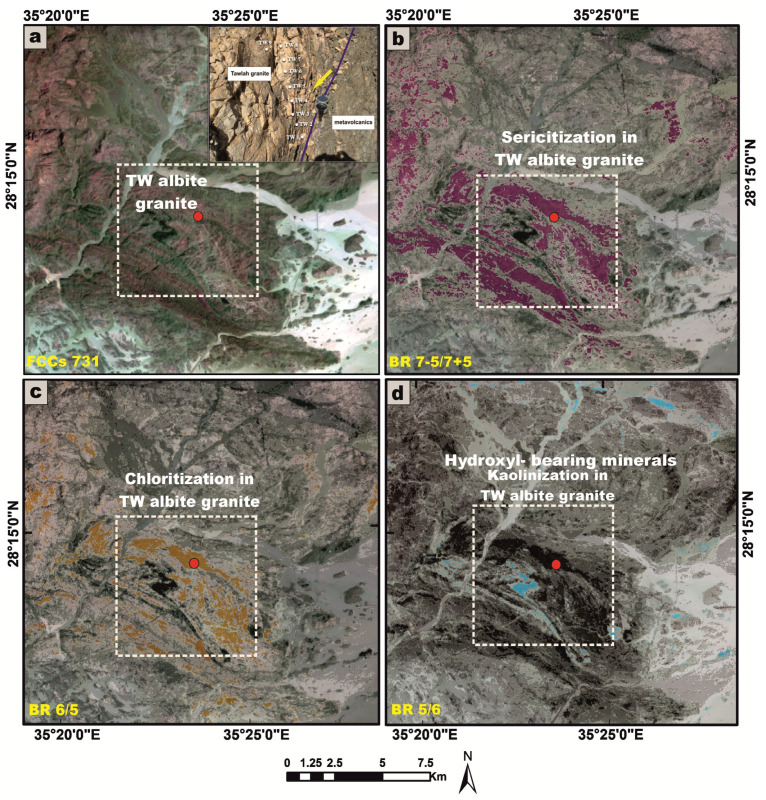
(**a**) Lithological discrimination using Landsat-9 FCCs 7-3-1 in RGB for TW albite granites; delineation and mapping of radioactive mineralization-bearing alteration zones associated with TW granites, utilizing Landsat-9 band ratios of (**b**) 7 − 5/7 + 5 for sericitization, (**c**) 6/5 for chloritization, and (**d**) 5/6 for kaolinization.

For TW granite, we used false-color composite band combinations (seven, three, and one in RGB) (Figure 23a) to provide good discrimination of the albite granite stock. To delineate alteration zones harboring radioactive mineralizations, advanced band ratio image processing techniques were applied with notable success. The band ratio (7 − 5)/(7 + 5) was instrumental in mapping sericitization alteration zones (Figure 23b). The band ratio 6/5 proved highly effective in identifying chloritization zones (Figure 23c), while the band ratio 5/6 proved highly effective in identifying hydroxyl-bearing minerals like kaolinite (kaolinization zones) (Figure 23d). We have already designated these locations by placing a white rectangular marker.

**Figure 24:**
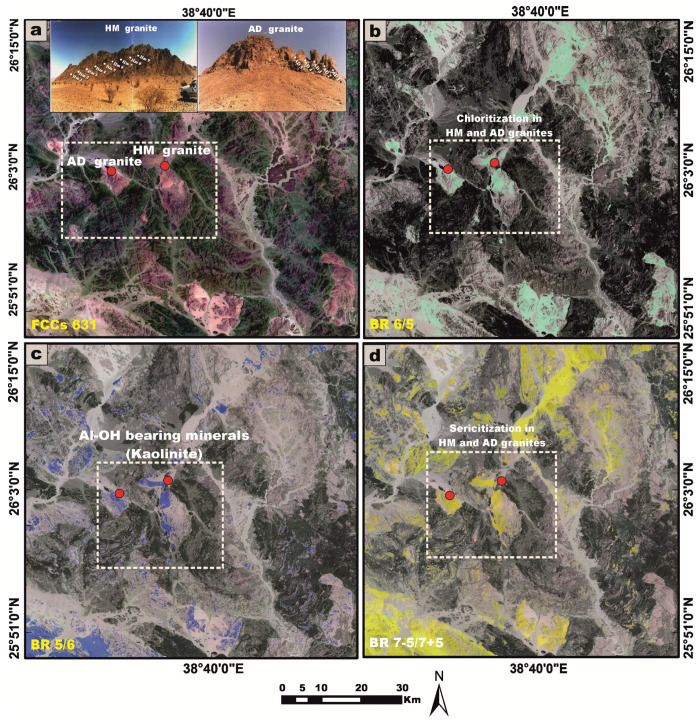
(**a**) Lithological discrimination using Landsat-9 FCCs 6-3-1 in RGB for HM alkali feldspar granites and AD granites; delineation and mapping of radioactive mineralization-bearing alteration zones associated with HM granites, utilizing Landsat-9 band ratios of (**b**) 6/5 for chloritization, (**c**) 5/6 for Al-OH bearing minerals (kaolinite and flouritization), and (**d**) 7 − 5/7 + 5 for sericitization.

For the HM and AD granites, FCC band combinations (six, three, and one in the RGB channels) were employed (Figure 24a). The same advanced image processing techniques as spectral band ratios were systematically applied for the effective identification of chloritization (Figure 24b), kaolinization (Figure 24c), and sericitization alteration zones (Figure 24d).

## 7. Conclusions and Recommendations

The concluding remarks synthesize the principal radiometric and geochemical characteristics governing the spatial distribution and provenance of natural radioactivity concentrations, specifically to ^238^U, ^226^Ra, ^232^Th, and ^40^K, within the granitic stocks of the Arabian Shield in Saudi Arabia, namely the TW, HM, and AD massifs.

**Figure 25:**
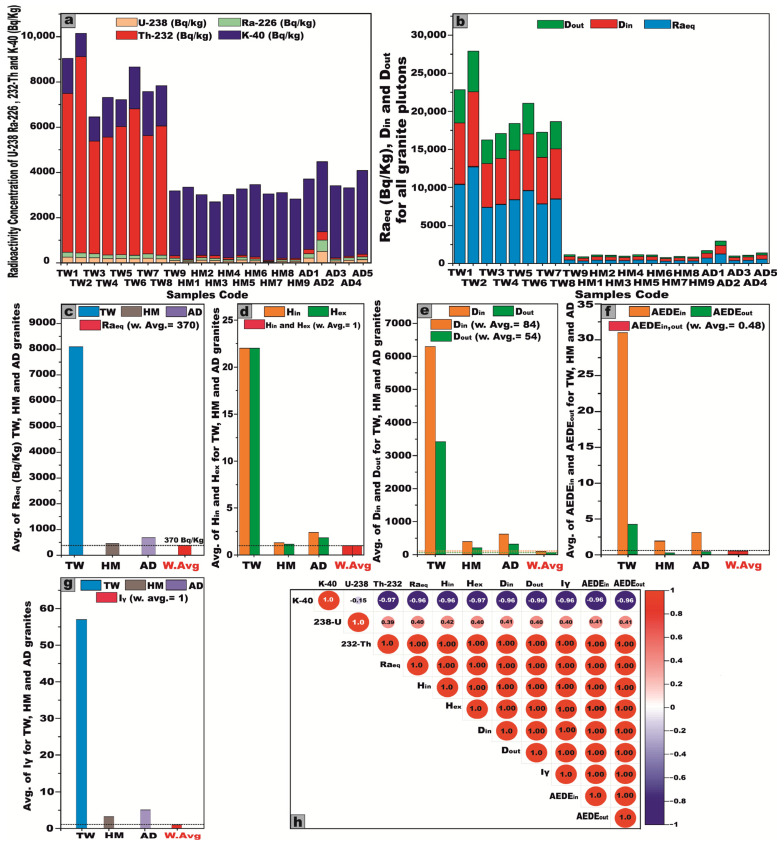
(**a**) Plot of the activity concentrations of radionuclide elements ^238^U, ^226^Ra, ^232^Th, and ^40^K (Bq/kg) using a stacked column diagram for TW, HM, and AD granitic plutons. (**b**) Plot of some radiological hazard factors (D_in_, D_out_, and Ra_eq_) using a stacked column diagram for all granitic plutons. A comparative assessment between the mean values of radiological hazard indices across all investigated granitic plutons and the globally recognized average levels: (**c**) for Ra_eq_ (Bq/kg), (**d**) for H_in_ and H_ex_, (**e**) for D_in_ and D_out_, (**f**) for AEDE_in_ and AEDE_out_, and (**g**) for I_γ_, and (**h**) Pearson correlation coefficients between radionuclide concentrations and associated radiological hazard indices. The dashed line indicates the internationally recognized average threshold.

The following conclusions are given below:

1. Laboratory-based radiometric analyses, performed using NaI (Tl) scintillation detectors, reveal that all examined granitic samples exhibit elevated concentrations of ^238^U and ^226^Ra, exceeding the globally accepted reference levels delineated by UNSCEAR (1993 and 2000) [1,5]. Furthermore, the samples display marked enrichment in ^232^Th and ^40^K, surpassing the international thresholds outlined in UNSCEAR (1993, 2000, and 2008) [1,5,27]. Moreover, it becomes evident that the dominant contributing radionuclide varies across the different granitic rock units, with each rock type exhibiting a disproportionate enrichment in a specific radioelement (Figure 25a,b).
2. All computed radiological hazard indices for the analyzed granitic samples generally exhibit values that surpass the internationally endorsed safety limits. Nonetheless, several noteworthy exceptions were identified: Samples HM1, HM7, and HM9 displayed Ra_eq_ and H_ex_ values that remained below the global average thresholds. Moreover, sample HM7 also exhibited a sub-threshold H_in_. Importantly, AEDE_out_ in sample TW9, as well as in all samples from the HM and AD stocks, consistently registered below internationally recognized reference values. However, upon examining the mean values of radiological hazard indices for each individual granitic rock unit, it becomes evident that the average levels generally exceed the global benchmarks, with the notable exception of the AEDE_out_ in the HM and AD stocks, which remain below the internationally accepted thresholds (Figure 25c–g). Hence, it is crucial to adopt a precautionary approach in any prospective mining or excavation activities within this region, necessitating increased vigilance and radiological awareness among personnel operating in adjacent mining sectors. Comprehensive risk mitigation strategies must be rigorously implemented to effectively monitor, manage, and minimize potential health hazards posed by elevated levels of natural radioactivity. Given the radiological burden observed in these granitic stocks, their utilization in construction materials or infrastructural applications is deemed unsuitable and potentially hazardous. Such materials should be categorically excluded from any form of human consumption or incorporation into inhabited structures to safeguard public health and environmental integrity.
3. In terms of geochemical datasets that confirm the conclusions based on radiometric measurements, the Th/U ratios reveal a pattern of significant uranium depletion in the TW granites, in contrast to the enrichment of uranium observed in the HM and AD stocks. This geochemical disparity is indicative of post-magmatic alteration processes, as depicted by using remote sensing datasets (Figure 23 and Figure 24). The AD granitic stock exhibits the highest average U and K concentrations. Conversely, the TW granites are distinguished by their notably elevated Th content, attributable to the abundance of radioactive accessory minerals such as thorite, sphene, zircon, and apatite. The positive correlations between U and Th across all granitic samples imply a common magmatic origin for these radionuclides, likely incorporated during the early crystallization phases of magma evolution.
4. A comparative assessment between radionuclide concentrations of U (Ra), Th, and K derived from chemical analysis and those obtained from radiometric measurements reveals that the latter consistently yields elevated average values for U and Th and vice versa for K across all granitic samples. This systematic discrepancy is indicative of post-magmatic uranium leaching and K enrichment.
5. Based on the calculated Pearson correlation coefficients, it is apparent that ^232^Th exhibits a strong positive correlation with all assessed radiological hazard indices, suggesting that it plays a predominant and influential role in governing the overall radiological profile of the studied granitic stocks (Figure 25h).
6. It is important to acknowledge that the distribution of U, Th, and K is strongly influenced by the mineralogical composition of the host rocks. While the present study provides comprehensive geochemical and radiometric data, the absence of quantitative mineralogical characterization introduces a degree of uncertainty when interpreting elemental correlations. So, the future studies incorporating detailed mineralogical analyses would enhance the interpretive framework and support a more robust understanding of the geochemical behavior of U, Th, and K in granitoid systems.

## Figures and Tables

**Table 1:** Specific activity concentrations of radioelements (^238^U, ^226^Ra,^232^Th, and ^40^K) and some ratios of studied granitic samples, Saudi Arabia, Arabian Shield.

Commercial Name		Activity Concentrations									
		U-238 Bq/kg	Ra-226 Bq/kg	Th-232 Bq/kg	K-40 Bq/kg	Th-232/Ra-226	Th-232/ K-40	Ra-226/K-40	Ra-226/Th-232	U-238/Th-232	U-238/K-40
TW1	Tawlah albite granite stock (TW)	252.8	220.0	7018.4	1550	31.9	4.5	0.14	0.03	0.04	0.16
TW2		230.5	210.1	8677.3	1028	41.3	8.4	0.20	0.02	0.03	0.22
TW3		221.3	182.9	4977.5	1070	27.2	4.7	0.17	0.04	0.04	0.21
TW4		184.7	165.2	5210.6	1750	31.5	2.9	0.09	0.03	0.04	0.11
TW5		192.65	171.2	5666.5	1189	33.1	4.8	0.14	0.03	0.03	0.16
TW6		179.3	155.7	6480.4	1844	41.6	3.5	0.08	0.02	0.03	0.09
TW7		207.4	192.7	5230.3	1950	27.1	2.7	0.09	0.04	0.04	0.11
TW8		180.2	158.9	5712.1	1779	35.9	3.2	0.08	0.03	0.03	0.10
TW9		108.6	94.6	111.2	2870	1.2	0.04	0.03	0.9	0.9	0.04
Min	Statistical Calculations	108.6	94.6	111.22	1028	1.18	0.04	0.03	0.02	0.03	0.04
Max		252.8	220.1	8677.3	2870	41.6	8.44	0.20	0.9	0.97	0.22
SD		38.7	34.6	2182.9	535.9	11.4	2.11	0.05	0.26	0.3	0.06
Avg.		195.3	172.4	5453.8	1670	30.1	3.9	0.12	0.12	0.14	0.13
HM1	Hamra alkali feldspar stock (HM)	70.1	56.1	34.44	3188	0.61	0.01	0.02	1.63	2.04	0.02
HM2		121.3	110.9	103.88	2677	0.94	0.04	0.04	1.07	1.17	0.05
HM3		105.8	93.5	121.76	2374	1.30	0.05	0.04	0.77	0.87	0.04
HM4		80.2	66.5	83.76	2788	1.26	0.03	0.02	0.79	0.96	0.03
HM5		131.2	117.7	89.66	2936	0.76	0.03	0.04	1.31	1.46	0.04
HM6		92.2	78.2	92.22	3199	1.18	0.03	0.02	0.85	0.99	0.03
HM7		44.1	32.2	42.33	2928	1.31	0.01	0.01	0.76	1.04	0.02
HM8		77.8	63.4	65.77	2899	1.04	0.02	0.02	0.96	1.18	0.03
HM9		75.2	62.1	60.65	2627	0.98	0.02	0.02	1.02	1.24	0.03
Min	Statistical Calculations	44.12	32.2	34.4	2374	0.6	0.01	0.01	0.76	0.9	0.02
Max		131.22	117.7	121.8	3199	1.31	0.05	0.04	1.63	2.03	0.05
SD		25.6	25.9	27.02	249.9	0.23	0.01	0.01	0.27	0.33	0.01
Avg.		88.66	75.62	77.16	2846.2	1.04	0.03	0.03	1.02	1.22	0.03
AD1	Abu al Dod pluton (AD)	210.1	194.33	192.4	3109	0.99	0.06	0.06	1	1.09	0.07
AD2		512.1	493.8	366.7	3100	0.74	0.12	0.16	1.35	1.39	0.17
AD3		88.1	74.12	55.7	3193	0.75	0.02	0.02	1.33	1.58	0.03
AD4		119.1	105.34	69.8	3023	0.66	0.02	0.03	1.51	1.71	0.04
AD5		142.1	124.4	116.5	3700	0.94	0.03	0.03	1.07	1.22	0.04
Min	Statistical Calculations	88.12	74.12	55.7	3023	0.66	0.02	0.02	1	1.09	0.03
Max		512.1	493.8	366.7	3700	0.99	0.12	0.16	1.51	1.71	0.17
SD		140.8	139.5	103.8	222.3	0.11	0.03	0.05	0.17	0.21	0.05
Avg.		214.3	198.4	160.2	3225	0.82	0.05	0.06	1.25	1.4	0.07

**Table 2:** Calculated environmental radiation hazard indices of studied granitic samples, Saudi Arabia, Arabian Shield.

Samples Name		U-238 (Bq/kg)	Ra-226 (Bq/kg)	Th-232 (Bq/kg)	K-40 (Bq/kg)	Ra_eq_ Bq/kg	H_in_	H_ex_	D (nGy/h)		AEDE (mSv/year)		I Index. (I_γ_)	ELCR	
									Indoor	Outdoor	Indoor	Outdoor		ELCR_in_ × 10^−3^	ELCR_out_ × 10^−3^
TW1	Tawlah albite granite stock (TW)	252.8	220.0	7018.4	1550	10,365.6	28.6	28.01	8049.8	4364.6	39.5	5.4	72.7	1.4	0.19
TW2		230.5	210.1	8677.3	1028	12,685.4	34.9	34.3	9822.6	5332.2	48.2	6.5	88.9	1.7	0.23
TW3		221.3	182.9	4977.5	1070	7375.9	20.4	19.9	5731.2	3105.9	28.1	3.81	51.71	0.98	0.13
TW4		184.7	165.2	5210.6	1750	7743.5	21.4	20.9	6027.1	3266	29.6	4	54.4	1.03	0.14
TW5		192.65	171.2	5666.5	1189	8357.6	23.1	22.6	6488.1	3518.4	31.8	4.32	58.6	1.11	0.15
TW6		179.3	155.7	6480.4	1844	9555.3	26.2	25.8	7422.9	4026.5	36.4	4.94	67.1	1.3	0.17
TW7		207.4	192.7	5230.3	1950	7814.6	21.6	21.12	6090.5	3298.3	29.9	4.04	54.9	1.05	0.14
TW8		180.2	158.9	5712.1	1779	8455.9	23.3	22.9	6575.4	3565	32.3	4.37	59.4	1.13	0.15
TW9		108.6	94.6	111.2	2870	474.21	1.54	1.3	444.7	227.5	2.18	0.28	3.7	0.1	0.01
Min	Statistical Calculations	108.6	94.6	111.22	1028	474.2	1.5	1.3	444.7	227.5	2.2	0.3	3.7	0.08	0.01
Max		252.8	220.1	8677.3	2870	12,685.4	34.9	34.3	9822.6	5332.2	48.2	6.5	88.9	1.7	0.23
SD		38.7	34.6	2182.9	535.9	3115.5	8.5	8.4	2393.7	1302.8	11.7	1.6	21.7	0.41	0.06
Avg.		195.3	172.4	5453.8	1670	8091.9	22.34	21.9	6294.7	3411.6	30.9	4.2	56.8	1.08	0.15
HM1	Hamra alkali feldspar stock (HM)	70.1	56.1	34.44	3188	350.6	1.1	0.9	350.9	178	1.7	0.21	2.8	0.06	0.01
HM2		121.3	110.9	103.88	2677	465.2	1.6	1.3	435.8	222.2	2.1	0.3	3.6	0.07	0.01
HM3		105.8	93.5	121.76	2374	450.1	1.5	1.2	414.7	212.7	2.03	0.26	3.4	0.07	0.01
HM4		80.2	66.5	83.76	2788	400.6	1.3	1.1	381.9	195.4	1.9	0.24	3.13	0.07	0.01
HM5		131.2	117.7	89.66	2936	471.6	1.6	1.3	447.6	227.4	2.2	0.28	3.6	0.07	0.01
HM6		92.2	78.2	92.22	3199	456	1.4	1.2	435.7	222.7	2.13	0.27	3.6	0.07	0.01
HM7		44.1	32.2	42.33	2928	317.9	0.9	0.9	316.3	161.5	1.6	0.19	2.6	0.06	0.01
HM8		77.8	63.4	65.77	2899	380.4	1.2	1	368.4	187.9	1.8	0.23	3.01	0.06	0.01
HM9		75.2	62.1	60.65	2627	350.8	1.1	0.9	339.3	172.9	1.7	0.21	2.8	0.06	0.01
Min	Statistical Calculations	44.12	32.2	34.4	2374	317.9	0.9	0.9	316.3	161.5	1.6	0.19	2.6	0.05	0.01
Max		131.22	117.7	121.8	3199	471.6	1.6	1.3	447.6	227.4	2.2	0.28	3.7	0.08	0.01
SD		25.6	25.9	27.02	249.9	54.6	0.2	0.15	44.9	22.9	0.2	0.03	0.37	0.01	0
Avg.		88.66	75.62	77.16	2846.2	404.8	1.3	1.1	387.8	197.9	1.9	0.24	3.17	0.07	0.01
AD1	Abu al Dod pluton (AD)	210.1	194.33	192.4	3109	708.4	2.4	1.9	645.4	329.6	3.2	0.4	2.3	0.11	0.01
AD2		512.1	493.8	366.7	3100	1256.1	4.7	3.4	1111.8	564.2	5.45	0.7	9.02	0.2	0.02
AD3		88.1	74.12	55.7	3193	399.3	1.3	1.1	391.2	198.8	1.9	0.24	3.2	0.1	0.01
AD4		119.1	105.34	69.8	3023	437.6	1.5	1.2	421.6	231.8	2.1	0.3	3.4	0.1	0.01
AD5		142.1	124.4	116.5	3700	575.4	1.9	1.6	545.9	278.3	2.7	0.34	4.5	0.1	0.01
Min	Statistical Calculations	88.12	74.12	55.7	3023	399.3	1.3	1.1	391.2	198.8	1.9	0.24	3.2	0.07	0.01
Max		512.1	493.8	366.7	3700	1256.1	4.7	3.4	1111.8	564.2	5.4	0.7	9.03	0.19	0.024
SD		140.8	139.5	103.8	222.3	283.2	1.14	0.8	237.9	120.681	1.2	0.15	1.9	0.04	0.01
Avg.		214.3	198.4	160.2	3225	675.3	2.4	1.8	623.2	316.9	3.1	0.4	5.1	0.11	0.01

**Table 3:** Calculation of the effective dose rate delivered to the organs (D_organ_).

Sample Names		AEDE Total	D (Lungs)	D (Ovaries)	D (Bone Marrow)	D (Testes)	D (Entire Body)
TW1	Tawlah albite granite stock (TW)	44.8	28.7	26	30.9	36.8	30.5
TW2		54.7	35.02	31.7	37.8	44.9	37.2
TW3		31.9	20.4	18.5	22.02	26.2	21.7
TW4		33.6	21.5	19.5	23.2	27.5	22.8
TW5		36.14	23.13	20.9	24.9	29.6	24.6
TW6		41.35	26.5	23.9	28.5	33.9	28.1
TW7		33.9	21.7	19.7	23.4	27.8	23.1
TW8		36.6	23.44	21.2	25.3	30.04	24.9
TW9		2.46	1.6	1.4	1.7	2.02	1.7
SD		13.34	8.53	7.73	9.2	10.9	9.1
Avg.		35.1	22.4	20.3	24.2	28.8	23.8
HM1	Hamra alkali feldspar stock (HM)	1.9	1.24	1.1	1.3	1.6	1.3
HM2		2.41	1.54	1.4	1.7	1.9	1.6
HM3		2.3	1.47	1.3	1.6	1.9	1.6
HM4		2.11	1.4	1.2	1.5	1.7	1.4
HM5		2.5	1.6	1.4	1.7	2.03	1.7
HM6		2.41	1.54	1.4	1.7	1.9	1.6
HM7		1.74	1.11	1.01	1.2	1.4	1.2
HM8		2.03	1.30	1.2	1.4	1.7	1.4
HM9		1.9	1.2	1.1	1.3	1.5	1.3
SD		0.25	0.16	0.14	0.17	0.20	0.17
Avg.		2.15	1.4	1.2	1.5	1.8	1.5
AD1	Abu al Dod pluton (AD)	3.6	2.3	2.1	2.5	2.9	2.4
AD2		6.15	3.9	3.6	4.24	5.04	4.2
AD3		2.16	1.4	1.3	1.5	1.8	1.5
AD4		2.33	1.5	1.4	1.6	1.9	1.6
AD5		3.02	1.9	1.8	2.1	2.5	2.1
SD		1.44	0.92	0.84	0.99	1.18	0.98
Avg.		3.44	2.20	1.9	2.4	2.8	2.34

**Table 4:** Comparison between ^238^U, ^226^Ra, ^232^Th, and ^40^K (Bq/kg) to some granitic and marble rocks in some countries around the world, with our studied granitic samples from Saudi Arabia, Arabian Shield.

Country	Avg ^226^Ra (Bq/kg)	Avg ^232^Th (Bq/kg)	Avg ^40^K (Bq/kg)	References
Nigeria	42.4	64.5	298	[47]
Turkey	47.5	43.7	487.0	[48]
Greece	51.4	22.6	134.2	[49]
Sudan	294.96	90.28	766.05	[24]
Nuba Mountain—Sudan	20.6	30.5	295.2	[24]
Jordan	20	11	85	[50]
Pakistan	33	32	57	[51]
China	90	116	969	[52]
India	119	172	1082	[53]
Brazil	131.6	285.8	1522.9	[54]
Holland	162	490	1540	[55]
USA	57	69	1140	[56]
Italy	112	107	1063	[57]
Algeria	55	--	410	[58]
Cameroon	8	0.4	19	[59]
Johor state—Malaysia	43.2	63.8	610.8	[60]
Iran	72	76	1193	[61]
Tawlah albite granite stock (TW)	172.4	5453.8	1670	
Hamra alkali feldspar stock (HM)	75.62	77.16	2846.2	
Abu al Dod pluton (AD)	198.4	160.2	3225	

**Table 5:** Comparison between ^226^Ra, ^232^Th, and ^40^K (Bq/kg) to some granitic and marble rocks in Egypt with our studied granitic samples from Saudi Arabia, Arabian Shield.

Area	Location	Avg ^226^Ra (Bq/kg)	Avg ^232^Th (Bq/kg)	Avg ^40^K (Bq/kg)	References
Wadi Ghazala	Southeastern Sinai	19	28.2	754	[62]
Wadi Sedri	Southwestern Sinai	33.3	33.47	403	[62]
Homret Mukpid	Eastern Desert	60.26	87.15	934	[62]
Igla	Eastern Desert	27.55	16.53	508	[62]
Zabara-Um Addebaa belt	Eastern Desert	24	6.73	2049	[62]
Commercial granitic (7 types)	-----------	55	51	1039	[63]
Mangual	Eastern Desert	78.4	84.4	903	[64]
El-Dib	Eastern Desert	74.9	94.3	1144.1	[64]
El-Urs	Eastern Desert	352	173.2	908.4	[64]
El-Risha	Eastern Desert	49.8	56.1	958.8	[64]
El-Qattar	Eastern Desert	104.4	78.8	892.9	[64]
Kab Amira	Eastern Desert	82	94.2	974.7	[64]
El-Gidami	Eastern Desert	169	134	951.5	[64]
Shalul	Eastern Desert	31.9	29	842.3	[64]
Bakreya	Eastern Desert	75	64.4	860.8	[64]
Sidi Salem	Eastern Desert	58.6	76.8	982.5	[64]
Mueilha	Eastern Desert	121.3	82.2	840	[64]
El-Sella	Eastern Desert	42.5	60.1	918.2	[64]
El-Misikat	Eastern Desert	1184	40	705	[65]
El-Eradiya	Eastern Desert	126	25	480	[65]
Abu Dabbab	Eastern Desert	46	20	602	[64]
El-Ruf -Monqul (NED)	Eastern Desert	38.32	47.19	992.26	[66]
Hafafit (SED)	Eastern Desert	30.8	27.3	1045.5	[67]
Black Aswan (SED)	Eastern Desert	29.6	44.4	803.37	[68]
Nero Aswan (SED)	Eastern Desert	25.6	55.21	855.53	
Red Aswan (SED)	Eastern Desert	44.40	92.92	1042.29	
Halayb (SED)	Eastern Desert	15.17	4.71	292	
Hurghada (NED)	Eastern Desert	111	86.19	939	
Karnak (CED)	Eastern Desert	55.5	46.46	616.61	
Verdi (SED)	Eastern Desert	49.95	44.44	968.74	
Abu Ramad Shear Zone (SED)	Eastern Desert	484.6	36.87	772.23	[69]
Tawlah albite granite stock (TW)	Saudi Arabia	172.4	5453.8	1670	
Hamra alkali feldspar stock (HM)	Saudi Arabia	75.62	77.16	2846.2	
Abu Al Dod pluton (AD)	Saudi Arabia	198.4	160.2	3225	

**Table 6:** Comparison between ^226^Ra, ^232^Th, ^40^K (Bq/kg), and some of the radiological parameters of some granitic rocks in Saudi Arabia with our studied granitic samples from Saudi Arabia, Arabian Shield.

Area	Avg ^226^Ra (Bq/kg)	Avg ^232^Th (Bq/kg)	Avg ^40^K (Bq/kg)	Ra_eq_ (Bq/kg)	D (nGy/h)	H_ex_	References
Qassim	23	30	340	61.89	25.08	0.17	[70]
Tabuk	24.35	35.39	375.96	103.91	139.3	0.28	[71]
Hai’l	102.46	486.75	725.95	844.46	9706.3	2.31	[72]
Riyadh	54.5	43.4	677.7	168.7	153	0.5	[73]
Study area TW	172.4	5453.8	1670	8091.9	9706.3	21.9	
Study area HM	75.62	77.16	2846.2	404.8	585.7	1.1	
Study area AD	198.4	160.2	3225	675.3	940.1	1.8	

**Table 7:** Geochemical analysis (XRF) for radioactive elements (U, Th ppm, and K_2_O w%) of studied granitic samples, Saudi Arabia, Arabian Shield.

Sample Name		U (ppm)	Th (ppm)	K_2_O (w%)	K (ppm)	U/Th	U-(Th/3.5)	Th/U	U-238 ppm (eU)	U_c_/U_r_ (D-Factor)
TW1	Tawlah albite granite stock (TW)	19.1	1522.7	3.1	30,800	0.01	−415.9	79.7	20.5	0.93
TW2		17.2	2124.8	2.4	24,200	0.01	−589.9	123.5	18.7	0.92
TW3		15.4	1225.1	2.5	24,700	0.01	−334.6	79.6	17.9	0.86
TW4		13.8	1282.2	2.1	20,900	0.01	−352.5	92.9	14.9	0.92
TW5		14.3	1393.3	2.6	26,300	0.01	−383.8	97.4	15.6	0.92
TW6		13.2	1594.4	2.2	22,100	0.01	−442.3	120.8	14.5	0.91
TW7		16.5	1286.5	2.3	23,400	0.01	−351.1	77.9	16.8	0.98
TW8		13.6	1406.6	2.1	21,300	0.01	−388.3	103.4	14.6	0.93
TW9		7.8	25.5	4.7	46,500	0.31	0.51	3.3	8.8	0.89
Min		7.8	25.5	2.09	20,900	0.01	−589.9	3.3	8.8	0.86
Max		19.1	2124.8	4.7	46,500	0.31	0.51	123.5	20.5	0.98
SD		3	523.3	0.76	7557	0.1	147.13	33.5	3.14	0.03
Avg.		14.5	1317.9	2.7	26,688.9	0.04	−361.9	86.5	15.8	0.92
HM1	Hamra alkali feldspar stock (HM)	4.5	7.4	5.02	50,200	0.61	2.4	1.6	5.7	0.79
HM2		9.3	24.1	4.4	44,200	0.39	2.4	2.6	9.8	0.95
HM3		7.7	28.5	4.1	40,500	0.27	−0.44	3.7	8.6	0.89
HM4		5.6	19.4	4.6	45,500	0.29	0.06	3.5	6.5	0.86
HM5		10	20.4	4.7	47,300	0.49	4.2	2.04	10.6	0.94
HM6		6.7	21	5.1	50,500	0.32	0.7	3.13	7.5	0.89
HM7		2.8	8.7	4.7	47,200	0.32	0.31	3.11	3.6	0.78
HM8		5.5	14.7	4.7	46,900	0.37	1.3	2.7	6.3	0.87
HM9		5.1	13.7	4.4	43,600	0.37	1.2	2.7	6.1	0.83
Min		2.8	7.4	4.1	40,500	0.3	−0.44	1.6	3.6	0.78
Max		10	28.5	5.1	50,500	0.61	4.17	3.7	10.6	0.95
SD		2.2	6.6	0.29	2998.7	0.1	1.4	0.62	2.16	0.1
Avg.		6.4	17.5	4.6	46,211	0.38	1.34	2.8	7.2	0.87
AD1	Abu Al Dod pluton (AD)	16.3	45.9	4.9	49,400	0.36	3.2	2.8	17.01	0.96
AD2		43.7	89.8	4.9	49,300	0.49	18.04	2.1	41.5	1.1
AD3		6.2	12.2	5.04	50,400	0.51	2.7	1.9	7.14	0.87
AD4		9.1	15.5	4.8	48,400	0.59	4.7	1.7	9.6	0.94
AD5		10.7	27.2	5.6	55,600	0.39	2.9	2.5	11.5	0.92
Min		6.2	12.2	4.84	48,400	0.36	2.7	1.7	7.14	0.87
Max		43.7	89.8	5.56	55,600	0.59	18.04	2.8	41.5	1.1
SD		13.7	28.4	0.3	2569.4	0.1	5.9	0.4	13.6	0.1
Avg.		17.2	38.1	5.1	50,620	0.47	6.31	2.2	17.4	0.95

**Table 8:** Transformation of radioelements (^238^U, ^226^Ra, ^232^Th, and ^40^K) from Bq/kg into ppm values.

Sample Name		U-238 Bq/kg	U-238 ppm (eU)	Ra-226 Bq/kg	Ra-226 ppm (eRa)	Th-232 Bq/kg	Th-232 ppm (eTh)	K-40 Bq/kg	K-40 ppm	eU −eTh/3.5	^238^U/^226^Ra (P-Factor)	eU/eRa (P-Factor)	eTh/eU
TW1	Tawlah albite granite stock (TW)	252.8	20.5	220.0	19.8	7018.4	1728.7	1550	4.9	−473.4	1.15	1.03	84.5
TW2		230.5	18.7	210.1	18.9	8677.3	2137.3	1028	3.3	−591.9	1.1	0.98	114.5
TW3		221.3	17.9	182.9	16.5	4977.5	1225.9	1070	3.4	−332.4	1.2	1.1	68.41
TW4		184.7	14.9	165.2	14.9	5210.6	1283.4	1750	2.6	−351.7	1.1	1	85.8
TW5		192.65	15.6	171.2	15.4	5666.5	1395.7	1189	3.8	−383.2	1.13	1.01	89.5
TW6		179.3	14.5	155.7	14.02	6480.4	1596.2	1844	5.9	−441.5	1.2	1.04	109.9
TW7		207.4	16.8	192.7	17.4	5230.3	1288.3	1950	6.2	−351.3	1.1	0.96	76.7
TW8		180.2	14.6	158.9	14.3	5712.1	1406.9	1779	5.7	−387.4	1.13	1.02	96.4
TW9		108.6	8.8	94.6	8.5	111.2	27.4	2870	9.1	0.96	1.15	1.03	3.1
Min		108.6	8.8	94.6	8.5	111.22	27.4	1028	3.3	−591.9	1.1	0.97	3.1
Max		252.8	20.5	220.1	19.8	8677.3	2137.3	2870	9.2	0.96	1.2	1.1	114.5
SD		38.7	3.13	34.6	3.11	2182.9	537.7	535.9	1.71	151.01	0.04	0.03	30.8
Avg.		195.3	15.8	172.4	15.5	5453.8	1343.3	1670	5.3	−367.9	1.13	1.02	80.9
HM1	Hamra alkali feldspar stock (HM)	70.1	5.7	56.1	5.1	34.44	8.5	3188	10.2	3.3	1.25	1.12	1.5
HM2		121.3	9.8	110.9	9.9	103.88	25.6	2677	8.6	2.5	1.1	0.98	2.6
HM3		105.8	8.6	93.5	8.4	121.76	29.9	2374	7.6	−0.01	1.1	1.02	3.5
HM4		80.2	6.5	66.5	5.9	83.76	20.6	2788	8.9	0.6	1.2	1.1	3.2
HM5		131.2	10.6	117.7	10.6	89.66	22.1	2936	9.4	4.3	1.1	1	2.1
HM6		92.2	7.5	78.2	7.05	92.22	22.7	3199	10.2	0.98	1.2	1.06	3.04
HM7		44.1	3.6	32.2	2.9	42.33	10.4	2928	9.4	0.59	1.4	1.23	2.9
HM8		77.8	6.3	63.4	5.7	65.77	16.2	2899	9.3	1.7	1.2	1.1	2.6
HM9		75.2	6.1	62.1	5.6	60.65	14.9	2627	8.4	1.8	1.2	1.1	2.5
Min		44.12	3.6	32.2	2.9	34.4	8.5	2374	7.6	−0.01	1.1	0.98	1.5
Max		131.22	10.6	117.7	10.6	121.8	29.9	3199	10.2	4.3	1.4	1.23	3.5
SD		25.6	2.1	25.9	2.32	27.02	6.7	249.9	0.79	1.3	0.08	0.1	0.6
Avg.		88.66	7.2	75.62	6.8	77.16	19	2846.2	9.1	1.7	1.2	1.1	2.6
AD1	Abu al Dod pluton (AD)	210.1	17.01	194.33	17.5	192.4	47.4	3109	9.9	3.5	1.1	0.97	2.8
AD2		512.1	41.5	493.8	44.5	366.7	90.3	3100	9.9	15.7	1.04	0.9	2.2
AD3		88.1	7.14	74.12	6.7	55.7	13.7	3193	10.2	3.2	1.2	1.1	1.9
AD4		119.1	9.6	105.34	9.5	69.8	17.2	3023	9.7	4.7	1.13	1.02	1.8
AD5		142.1	11.5	124.4	11.2	116.5	28.7	3700	11.8	3.3	1.14	1.03	2.5
Min		88.12	7.1	74.12	6.7	55.7	13.7	3023	9.7	3.2	1.04	0.93	1.8
Max		512.1	41.5	493.8	44.5	366.7	90.3	3700	11.8	15.7	1.2	1.1	2.8
SD		154.2	12.5	152.9	13.8	113.7	28.01	243.5	0.78	4.8	0.05	0.05	0.36
Avg.		214.3	17.4	198.4	17.9	160.2	39.5	3225	10.3	6.1	1.1	1	2.2

N.B: 1 ppm of U-238 = 12.35 Bq/kg, 1 ppm of Ra-226 = 11.1 Bq/kg, 1 ppm of Th-232 = 4.06 Bq/kg, and 1 ppm of K-40 = 313 Bq/kg [74].

## Data Availability

Data are contained within this article. The original contributions presented in this study are included in this article. Further inquiries can be directed to the corresponding author.
